# Acknowledgment to Reviewers of *Polymers* in 2020

**DOI:** 10.3390/polym13030350

**Published:** 2021-01-22

**Authors:** Polymers Editorial Office

**Affiliations:** MDPI AG, St. Alban-Anlage 66, 4052 Basel, Switzerland

Peer review is the driving force of journal development, and reviewers are gatekeepers who ensure that *Polymers* maintains its standards for the high quality of its published papers. Thanks to the cooperation of our reviewers, in 2020, the median time to first decision was 11.5 days and the median time to publication was 31 days. The editors would like to express their sincere gratitude to the following reviewers for their precious time and dedication, regardless of whether the papers were finally published:
Abate, LorenzoLou, Ching-WenAbbasi, RezaLou, Shi NeeAbbes, BoussadLou, TiejiongAbbrent, SabinaLou, YanAbdel-Fattah, EssamLou, ZhichaoAbd-Elnaiem, AlaaLovestead, TaraAbdelsalam, Sara I.Lowry, MarkAbdo, HanyLozano Guerrero, Antonio JoséAbedini-Nassab, RoozbehLozinsky, VladimirAbejón, RicardoLu, PengtaoAbenojar, JuanaLu, Qing-HuaAbramović, BornaLu, WeiAbuin, GracielaLu, XinyiAbushammala, HatemLu, YangfanAccorsi, GianlucaLubczak, JacekAcharya, GhanashyamLucaciu, Constantin MihaiAchilias, Dimitris S.Lucchetta, GiovanniAdachi, NaoyaLuciani, GiuseppinaAdamczyk, GretaLuciano, GiorgioAdams, MikeLuckham, PaulAdamus, GrazynaLuda, Maria PaolaAddiego, FredericLukasik, RafalAdera, SolomonLukomska-Szymanska, MonikaAdhikari, Birendra B.Lukyanov, Pavel A.Adhikary, AmitavaLuliński, SergiuszAdomavičiūtė, ErikaLuna, CarlosAdonin, Sergey A.Lund, RafaelAdumitroaie, AdiLundin, Jeffrey G.Aflori, MagdalenaLunguleasa, AurelAfzal, AdeelLunt, Alexander J.G.Agarski, BorisLuo, JunAgatemor, ChristianLuo, KuiAguiar, AndréLuo, LeiAguiar, Monica LopesLuo, RongcongAguilar-Bolados, HectorLuo, RuiyingAguirre-Álvarez, GabrielLuo, Sheng-NianAhad, AbdulLuo, YanhuaAhmad, JavedLuo, YuanfangAhmad, Mohd ZamidiLuo, YunjunAhmadivand, ArashLützenkirchen, JohannesAhmed, MaryaLuyt, AdriaanAhmed, Sheikh AliLuz, SandraAhmmed, K.M. TanvirLuzi, FrancescaAhsan, Md. ArifulLv, HaifeiAida Alejandra, Pérez-FonsecaLyu, HailongAirey, GordonLyulin, Alexey V.Airoudj, AissamM. Gómez, ClaraAissa, BrahimM. Wallner, GernotAjmal, Hafiz Muhammad SalmanMa, FuyingAkhremitchev, Boris B.Ma, JiangyaAkita, SadanoriMa, JianzhongAkitaya, TatsuoMa, LiangAkitsu, TakashiroMa, SongqiAkitsu, TetsuyaMacagnano, AntonellaAkopova, TatianaMachado, FabricioAl-Anssari, SarmadMaciejewska, MagdalenaAlbo, JonathanMadbouly, SamyAlcoutlabi, MatazMadurga, SergioAldalbahi, AliMady, Carlos Eduardo KeutenedjianAlexandrova, LarissaMaeda, TomokiAlexandru, AsandeiMaeyama, TakuyaAlfano, MarcoMagalhães, Fernão D.Alfei, SilvanaMagalhães, SolangeAlhwaige, AlmahdiMagdalena, StepczynskaAli Khan, MoonisMagkoev, Tamerlan T.Ali Shah, Anwar-ul-HaqMaisetta, GiuseppantonioAllais, FlorentMaitland, DuncanAllen, Norman S.Majee, SubimalAlmazán Lázaro, Juan AntonioMajer, JanjaAlmeida Júnior, José HumbertoMajewski, PawelAlmoazen, HassenMaji, TanmoyAlnaser, Ali S.Majoinen, JohannaAlosmanov, RasimMakshakova, OlgaAlswieleh, Abdullah M.Maksimkin, AlekseyAlt, HelmutMaksoud, TalalAltan, M. CengizMakvandi, PooyanAltarawneh, MohammednoorMalara, AngelaAltarawneh, Mohammednoor KhalilMaldonado, José-LuisAlvarado, VladimirMałecka, MagdalenaAlvarez, Alejandro J.Malik, Muhammad ImranAlvarez, MarMalinowski, RafałAlvarez-Idaboy, Juan RaùlMalkin, AlexanderAlvarez-Malmagro, JuliaMallamace, DomenicoAlvarez-Ramirez, JoseMallamace, FrancescoAlves, LuisMałolepszy, ArturAlves, NunoMalta, Luiz F. B.Amante, Edna ReginaMalucelli, GiulioAmariei, SoniaMamajanov, IrenaAmbrosio-lazaro, Roberto CarlosMamidi, NarmidiAmendola, EugenioManaila, ElenaAmer, HassanManakhov, AntonAmii, HidekiMandal, MrinmayAmine, AzizManfredi, AmedeaAn, HyosungManfredini, StefanoAn, SeongpilMangalara, Jayachandra HariAnanth, AntonyMangwandi, ChiranganoAnanthakrishnan, Soundaram JeevarathinamMani, Ganesh KumarAndia, IsabelManian, Avinash P.Andreoli, EnricoManjakkal, LibuAndreopoulou, Aikaterini K.Mannheim, ViktoriaAndronic, LuminitaManouras, TheodorosAndrzejewski, JacekMantione, DanieleAng, Micah Belle Marie YapMantovani, GiuseppeAngelopoulou, AthinaMaraveas, ChrysanthosAnis, BadawiMarciniec, BogdanAnitas, EugenMarcinkowska, AgnieszkaAnnamalai, Pratheep KumarMarconcini, PaoloAnraku, MakotoMarcovich, Norma E.Antal, DianaMareev, E. I.Anthamatten, MitchellMarega, CarlaAntónio Correia, JoséMarić, MilanAntonioli, DiegoMarin, LuminitaAntov, PetarMarin, Ricardo MallaviaAntunes, AnaMarín-Genescà, MarcAntunes, Joana C.Marinkovic, AdAntunes, MarceloMarotti De Sciarra, FrancescoAntunes, VanessaMarpu, Sreekar BabuAnugwom, IkennaMarras, AlexanderAnyszka, RafałMarrocchi, AssuntaAoki, HiroyukiMartikka, OssiAparicio, Francisco J.Martin, PatrickApel, Pavel Y.Martin, SimonApetrei, ConstantinMartinelli, AndreaApostolidis, PanosMartinelli, EnzoAraby, SherifMartínez De Ilarduya, AntxonAranguren, Mirta I.Martínez López, José IsraelAraújo, EvandoMartinez, CristinaAraya-Hermosilla, RodrigoMartínez-Espinosa, Rosa MaríaArdelean, Lavinia CosminaMartínez-López, Ana LuisaArena, MaurizioMartínez-Palou, RafaelArifuzzaman, MohammadMartínez-Pérez, Carlos A.Ariga, KatsuhikoMartínez-Tong, Daniel E.Arinstein, ArkadiMartinho, José GasparArjmandi, RezaMartini, FrancescaArkas, MichaelMartinka, JozefArnould, MarkMartinotti, SimonaArrabito, Giuseppe DomenicoMartín-Ramos, PabloArrieta, MarinaMartín-Rapún, RafaelArrieta-Baez, DanielMartins Amaro, Ana Paula BetencourtArrigo, RossellaMartins, Luis R. M.Arunachalam, PrabhakarnMartins, RobertoAsandulesa, MihaiMaruda, Radosław W.Ashley, JonMaruyama, KeiAshourirad, BabakMasania, KunalAssaf, Khaleel IbrahimMasato, DavideAsuri, PrashanthMascia, LenoAta, SeisukeMasek, AnnaAtabaev, Timur Sh.Mashaan, NuhaAtanase, LeonardMasłoń, AdamAthanasiadis, VassilisMassoud, SalahAthanasiou, Christos-EdwardMasuda, YoshitakeAtif, RasheedMasullo, MariorosarioAtrei, AndreaMatei, AlinaAtta-Obeng, EmmanuelMatei, CristianAttri, PankajMateo Fernandez, SaraAugustyniak, AdrianMatharu, Rupy KaurAuriemma, FiniziaMatko, VojkoAvila-Orta, Carlos AlbertoMatsidik, RukiyaAvilés, M.D.Matsui, JunAvolio, RobertoMatsumoto, KozoAwual, Md. RabiulMatsumoto, ToshihikoAyre, DavidMatsumura, ShuichiAyres, NeilMatykiewicz, DanutaAytemiz, DeryaMaurício Alves, Da Motta SobrinhoAyyavoo, JayalakshmiMautner, AndreasAzevedo, AfonsoMaver, TinaAzimi, BaharehMavrantzas, VlasisB.C. Pergher, SibeleMawatari, YasuteruBaba, KoumeiMay, MichaelBabarao, RavichandarMazinani, SaeedBabu, Libin K.Mazzaglia, AntoninoBácskay, IldikóMazzanti, ValentinaBadea, MihaelaMcGuinness, GarrettBadia, JoseMechrez, GuyBadilescu, SimonaMedellín-Rodríguez, Francisco J.Badshah, Mohsin AliMederic, PascalBae, Byung-ChanMedina-Ramírez, IlianaBae, Hae-RahnMeenashisundaram, Ganesh KumarBae, Ki HyunMees, MaartenBaek, YoungbinMehrali, MehdiBagheri, AliMei, HanfeiBagi, PéterMei, HuiBaglioni, MicheleMei, LanjuBahk, Je-HyeongMeier, Robert J.Bahmani, AramMejia-Beltran, EfrainBai, ChengyingMelinte, VioletaBai, ChenxiMemon, HafeezullahBai, DongyuMendes, AdrianoBai, HongweiMéndez, José AlbertoBai, JiamingMeneguzzi, AlvaroBai, RuixiangMeng, FanbinBai, YangMeng, HongBajpai, AnkurMeng, ShujuanBakar, MohamedMeng, XiangchaoBakhshian, SaharMenger, Fredric M.Balach, JuanMengistie, Desalegn AlemuBalandin, Alexander A.Menon, DeepthyBalashov, Victor N.Menoufi, KarimBalcar, HynekMensik, MiroslavBaldino, LuciaMequanint, KibretBaljon, Arlette R. C.Merabia, SamyBallard, NicholasMercado-Colmenero, Jorge ManuelBaloukas, BillMesquita, LuisBaltriukiene, DaivaMessori, LuigiBalzer, FrankMetón, IsidoroBambach, MikeMézes, MiklósBanaszak, SzymonMezzi, AlessioBandari, SureshMezzomo Collares, FabrícioBandi, RajkumarMhlongo, Gugu H.Banerjee, Shib ShankarMi, DashanBao, YinyinMiao, DagangBaptista, Cristina M. S. G.Miao, QingqingBarabanova, Anna I.Michael, AronBarandiaran, IratiMichihata, MasakiBaranowska, HannaMichl, ThomasBárány, TamásMichna, AnetaBarata, Joana F. B.Miculescu, FlorinBarati, DanialMicutz, MarinBarbalexis, PanagiotisMigita, SatoshiBarbera, JoaquinMihai, MaraBarberis, FabrizioMihai, MihaelaBarbinta-Patrascu, M. E.Mija, AliceBarczewski, MateuszMijović, BudimirBardosova, MariaMikeš, PetrBarille, RegisMikulchyk, TatsianaBarjas, Gustavo CabreraMilani, GabrieleBarman, Barun KumarMilasius, RimvydasBarnat-Hunek, DanutaMilchev, AndreyBarra, Guilherme M.O.Milčius, DariusBarrett, Stephanie E.Mileo, PauloBarretta, RaffaeleMiller, ReinhardBarroca, Maria JoãoMills, DavidBarros Gonçalves, Luciana RochaMiluski, PiotrBarry, CarolMin, JunyingBarszczewska-Rybarek, IzabelaMin, RuiBaschieri, AndreaMinak, GiangiacomoBasinska, TeresaMinakshi, ManickamBasoli, FrancescoMinami, IchiroBassetti, MauroMincheva, RosicaBassyouni, MohamedMinea, Alina AdrianaBattegazzore, DanieleMinelli, MatteoBattistel, Postdoc AlbertoMinoda, MasahikoBattocchio, ChiaraMiquelard-Garnier, GuillaumeBatys, PiotrMiranda, Jose M.Bauer, FrancoisMiri, Amir K.Baumgarten, MartinMirkhani, Seyyed AlirezaBayer, IlkerMiroslaw, WesolowskiBayraktar, EminMirsayar, MirMiladBazilevskaya, KatyaMirski, RadosławBecker, DanielaMirvakili, Seyed M.Bedini, EmilianoMishra, Geetesh K.Bednarz, SzczepanMishra, Pawan KumarBehnood, AliMishra, YogendraBekhta, PavloMisiakos, KonstantinosBełdowski, PiotrMissinne, JeroenBell, Carl-MartinMistewicz, KrystianBella, FedericoMiszczyk, AndrzejBellantone, VincenzoMitchell, GeoffreyBellisario, DeniseMitchell, Scott G.Bellotto, MaurizioMitropoulos, Alexander N.Beltsios, KonstantinosMitropoulos, Athanasios C.Benedetti, Assis VicenteMitsumata, TetsuBeneš, HynekMittal, NiteshBenesperi, IacopoMitu, BogdanaBenetatos, PanayotisMiyazaki, ToshikiBengtson, GiselaMizoshita, NorihiroBenito, Juan M.Mizusaki, MasanobuBenková, ZuzanaMizzi, LukeBermeshev, MaximMoad, GraemeBernardo, Carlos A. A.Moatsou, DafniBernards, MatthewMobed-Miremadi, MaryamBernasconi, RobertoModigell, MichaelBernhard, YannModukuri, RamkumarBernkop-Schnürch, AndreasMoggio, IvanaBertacchini, JessikaMohammadi, FazelBertani, RobertaMohammadiarani, HosseinBerteau, Jean-PhilippeMohammed, Abdul SamadBerton, NicolasMohammed, NishilBesford, QuinnMohan, Velram BalajiBeskopylny, AlexeyMohles, VolkerBettotti, PaoloMojiri, AminBeuermann, SabineMokhtari, FatemehBhang, Suk HoMołczan, MarekBharath, Kurki NagarajaMolina, MariaBhatia, DivijMollica, FrancescoBhattacharya, SupriyoMondal, GoutamBhattarai, AjayaMondal, KunalBhatti, M.MMonobe, HirosatoBhudolia, Somen K.Montalto, LuigiBi, KeMontastruc, LudovicBiałek, MarzenaMonteil-Rivera, FannyBian, HuiyangMonteiro, Sergio NevesBian, LimingMonti, MarcoBianchi, MassimilianoMoo, Eng KuanBielas, RafałMoon, BongjinBielawski, Christopher W.Moon, Hong ChulBieliński, Dariusz M.Moraczewski, KrzysztofBiernat, JanMorales-Morales, DavidBikiaris, DimitriosMorales-Rojas, HugoBilal, SalmaMorallon, EmiliaBildirir, HakanMoramarco, VincenzoBilisik, K.Morazzoni, FrancaBîrleanu, Corina J.Moreirinha, CatarinaBisbey, RyanMoreno, Angel JBiscaia, Hugo C.Morgado, JorgeBisinella, Radla Zabian BassettoMorgan, AlexanderBiswas, ChandanMori, WasukeBitsanis, IoannisMorikawa, AtsushiBittolo Bon, SilviaMorimura, ShigeruBlanco, AngelesMoro Piekarski, CassianoBlanco, IgnazioMorovati, VahidBlencowe, AntonMorreale, MarcoBobbala, SharanMorris, Michael A.Bobis-Wozowicz, SylwiaMorselli, DavideBobrovsky, AlexeyMort, AndrewBocahut, AnthonyMoschopoulou, EkateriniBoccia, Antonella CaterinaMosleh, Ahmed O.Boda, Sunil KumarMostazo-López, María JoséBodaghi, MahdiMotoyanagi, JinBodoki, EdeMotra, HemBoesel, LucianoMourzenko, ValeriBoffa, VittorioMoustafine, RouslanBogdan, CatalinaMovafaghi, SanliBogdan, TutunaruMozejko-Ciesielska, JustynaBoggioni, LauraMozumder, Mohammad SayemBöhm, MichałMrlík, MiroslavBohm, SivasambuMrówczyński, RadosławBokias, GeorgiosMrukiewicz, MateuszBollini, FlaviaMu, Hai-JinBolotov, LeonidMu, RuipuBonadies, IreneMucci, VeronicaBonartsev, AntonMujtaba, MuhammadBonilla-Cruz, JoséMukherjee, SantanuBonvent, Jean-JacquesMukherjee, SudipBorges, João PauloMukhin, NikolayBoris, PejinMunoz, MiguelBorisov, OlegMunoz-Pinto, DanyBormashenko, EdwardMunteanu, Florentina-DanielaBoroń-Kaczmarska, AnnaMurai, ShunsukeBorowicz, MarcinMurawski, CarolineBoschetto, FrancescoMurshid, NimerBotelho, GabrielaMurugappan, KrishnanBou, Santiago FerrandizMusat, VioricaBoucher, David S.Musioł, MartaBourbigot, SergeMustata, Fanica R.Bousek, JaroslavMusuc, Adina MagdalenaBoyarskiy, Vadim P.Muthu Karuppan, Mohan KumarBraatz, RichardMuthuraj, RajendranBracciale, Maria PaolaMutjè, PereBrancart, JoostMutlu, HaticeBranda, FrancescoMutreja, IshaBrant, DavidMyler, PeterBratek-Skicki, AnnaMysiukiewicz, OlgaBrauner, EdoardoMyśliwiec, JarosławBrebu, MihaiMystkowska, JoannaBrecker, LotharNa, Jun-HeeBrennan, CharlesNa, LiBriatico-Vangosa, FrancescoNabil, BouaziziBrischetto, SalvatoreNaccarato, AttilioBroda, JanNadolny, ZbigniewBroda, MagdalenaNag, AnindyaBrosseau, ChristianNagamine, KuniakiBrostow, WitoldNagane, SatyawanBrunel, FabriceNagel, JürgenBrunella, ValentinaNagy, EndreBrunner, AndreasNair, AshwinBruno, RosariaNair, Sandeep SudhakaranBruns, Carson J.Naito, KatsuyukiBruns, NicoNakabayashi, KojiBrzeska, JoannaNakaoki, TakahikoBrzezinski, MarekNakas, James P.Brzózka, AgnieszkaNakata, NorioBuaki-Sogó, MireiaNakayama, YasuyaBubnov, AlexeyNakayama, YuushouBucio, EmilioNam, Sang YongBuckner, Steven WNamjoshi, SarikaBucur, BogdanNand, AshveenBudinski-Simendić, JaroslavaNaolou, ToufikBuj-Corral, IreneNarducci, RiccardoBulboacă, Adriana ElenaNarita, FumioBulgariu, LauraNaruta, YoshinoriBunoiu, Octavian MădălinNascimento, Luís Adriano Santos DoBuonomenna, Maria GiovannaNaser, M. Z.Burmistrov, IgorNash, Michael A.Bury, WojciechNastuta, Andrei VasileBustillos, JennifferNatalello, AntoninoButu, AlinaNath, ArijitByun, MyunghwanNatkański, PiotrCaballero, AntonioNaumov, Anton V.Cabanelas Valcárcel, Juan CarlosNaushad, MCabrales, LuisNaushad, Mu.Cacciotti, IlariaNavarro, Francisco JavierCadinoiu, Anca NiculinaNavarro, RodrigoCaggiano, AntonioNawaz, MuhammadCai, JinguangNaylor, AndrewÇakmakçi, EmrahNazare, ShonaliCalabrò, Paolo S.Nazir, RashidCalamak, SemihNeagu, AdrianCaldas, PauloNechifor, GheorgheCalderón, VerónicaNeel, Ensanya A. AbouCalì, MicheleNegahdar, LeilaCallejas, AntonioNegut, IrinaCalvo, LourdesNeighbour, GarethCamaiti, MaraNeisiany, Rasoul EsmaeelyCamba, A. TundidorNelatury, SudarshanCametti, CesareNemes, Norbert M.Caminero Torija, Miguel ÁngelNerin, CristinaCampiglio, Chiara EmmaNeto, MariaCampíns-Falcó, PilarNetrusov, AlexanderCampos Rubio, Juan CarlosNetz, PauloCañavate, JavierNeugebauer, DorotaCandan, ZekiNevárez-Moorillón, Guadalupe VirginiaCandau, NicolasNeves, RuiCanela-Garayoa, RamonNg, Wei LongCanestrari, FrancescoNgai, ToCangialosi, DanieleNghia, Truong PhuocCao, BingNgo, Tri DungCao, JunNgo, Tri-DungCao, WeiNguyen, Duc ThanhCao, XiaodongNguyen, KateCapacchione, CarmineNguyen, Ngoc A.Capasso, RaffaeleNguyen, Tuan AnhCapezza, AntonioNicasio, Maria CarmenCaputo, PaolinoNicoletta, Fiore PasqualeCaraschi, José ClaudioNie, JunCarbone, AlessandraNie, YijingCárdenes, VíctorNiemczak, MichalCardon, LudwigNiemczyk, AgataCaridad, JoseNieminen, KaarloCarmen Sánchez, María DelNieto García, DanielCarmona-Ribeiro, Ana M.Nievergelt, Adrian PascalCarosio, FedericoNiidome, TakuroCarraro, MauroNikolaidis, Alexandros K.Carroccio, Sabrina CarolaNikolakakis, IoannisCarter, AlanNikoleli, Dimitrios P.Carvajal-Millan, ElizabethNikolic, LjubisaCarvalho, LuisaNinarello, AndreaCarvalho, Luisa M.H.Nine, Md JulkerCarvalho, Nakédia M.F.Ning, HaibinCasado-Coterillo, ClaraNing, HuimingCaschera, DanielaNishikitani, YoshinoriCasimiro, Maria HelenaNishitsuji, ShotaroČáslavský, JosefNisticò, RobertoCastell, PereNistor, CristinaCastiglione, FrancaNita, Loredana E.Castorena-Torres, FabiolaNitschke, MirkoCastro, Mariana S.Nitulescu, MihaiCastro-Muñoz, RobertoNiu, HaijunCatanzano, OvidioNjuguna, JamesCatapano, AnitaNoack, JensCatauro, MichelinaNobile, LucioCatenacci, LauraNoman, Muhammad TayyabCavallaro, GiuseppeNomngongo, Philiswa NosizoCavallo, DarioNoor, NuruzzamanCavalu, SimonaNoordergraaf, Inge-WillemCavicchi, KevinNorek, MałgorzataCazacu, AnaNouvel, CécileCeccone, GiacomoNovak, SanjaCech, VladimirNowacka, MariaCecilia, Juan AntonioNowaczyk, JacekCefalas, Alciviadis-ConstantinosNowicki, MichałCeglowski, MichalNsooudi, Nasim NsooudiCennamo, NunzioNtetsikas, KonstantinosCerbu, CameliaNumez, EstrellaCerna, JorgeNunes, MartaČernoch, PeterNunes-Pereira, J.Cerrada, Maria L.Nys, IngeCerri, Marcel OtavioOancea, FlorinCerrone, FedericoObata, KotaroCerroni, BarbaraOchiai, BungoCerruti, PierfrancescoOchoa, Nelio ArielČervenka, MilanOgale, Amod A.Cerveny, SilvinaOgunsona, EmmanuelCesano, FedericoOh, Kyung WhaCeseracciu, LucaOhara, KeishiCevik, EmreOhira, AkihiroCha, ChaenyungOhkita, HideoCha, Sang-HoOikonomou, Evdokia K.Cha, Sung WoonOjeda-Benitez, SaraChacón Muñoz, Jesús MiguelOkazaki, ToshiyaChae, MichaelOkubo, TakashiChakrabarty, ArindamOlad, AliChalioris, ConstantinOlar, RodicaChan, Yi-TsuOlariu, Marius-AndreiChandrasekaran, MurugesanOlewnik-Kruszkowska, EwaChang, Boon PengOliveira, Victor Manuel BarbasChang, Che-ChenOlyaee, SaeedChang, Chih-WeiOmbres, LucianoChang, Dong WookOmiya, MasakiChang, Ji WoongOnuta, Marie-ChristineChang, JiyoungOpran, ConstantinChang, MincheolOreshonkov, AleksandrChang, ShuaiOrooji, YasinChang, Young-WookOrozco, JahirChangzeng, YanOrtega, ZaidaChao, HuikuanOrtega-Lara, WendyChapel, Jean PaulOrtyl, JoannaCharas, AnaOsajima, Josy A.Charcosset, CatherineOsakada, KohtaroCharvatova, HanaOśmiałowski, BorysChatterjee, SudiptaOsmulski, Pawel A.Chaubet, FrédéricOsses, NelsonChaukura, NhamoOsswald, TimChavez-Angel, EmigdioOstrauskaite, JolitaChee Kai, ChuaOstrowski, KrzysztofCheimarios, Nikolaos (Nikos)Ostrowski, Krzysztof AdamChen, ChangleOtero-Irurueta, GonzaloChen, ChengOtoni, Caio GomideChen, Cheng-HoOubaha, MohamedChen, ChiachungOuellet-Plamondon, ClaudianeChen, Chi-TienOulego, PaulaChen, Chun-ChiOuyang, Xiao-kunChen, Chun-LongOwczarz, PiotrChen, DazhengPachapur, VinayakChen, DongyangPacheco, NeithChen, FumingPacheco-Catalán, DaniellaChen, GuangmingPaciorek-Sadowska, JoannaChen, HaoPadil, Vinod V. T.Chen, Hsuan-YingPadilla, JavierChen, Jeff Z.Y.Pagacz, JoannaChen, Jem-KunPagani, StefaniaChen, Jian-LianPaganin, Valdecir AntonioChen, JingPagano, StefanoChen, JinhuaPaiè, PetraChen, JunPalacio, LauraChen, Jung-YaoPalamà, IlariaChen, Jyh-PingPalchesko, RachelleChen, LingPalermo, EdmundChen, LingxinPall, EmokeChen, Lung-ChienPalla, FrancoChen, PengPalma, Paulo JorgeChen, Roland K.Palmieri, Frank L.Chen, RongjunPalmieri, ValentinaChen, San-YuanPaltanea, GheorgheChen, Shih-YunPaluch, Andrew S.Chen, XiaoyiPalumbo, CarlaChen, Yi-ChunPalumbo, FabioChen, Yi-WenPalumbo, GaetanoChen, ZhihangPalumbo, OrieleChen, ZhongbingPamies, RamonCheng, Chih-ChiaPan, GuoqinCheng, Huai N.Pan, GuoqingCheng, LishengPan, HaihuaCheng, PeiPan, YangangCheng, QilinPan, Yi-JunCheng, ZhengdongPan, YuanfengCheong, In WooPanaitescu, Denis MihaelaCheong, Kit LeongPanda, BiranchiCheraghi Bidsorkhi, HosseinPandele, MadalinaCheraghian, GoshtaspPandey, SadanandCheskis, DimaPánek, MilošChevali, VenkataPang, JinhuiChi, HongPanin, SergeyChiang, Chih-hungPanizza, MatteoChiang, Chin LungPanomsuwan, GasiditChiang, Yi-TingPant, BishweshwarChiao, MuPantani, RobertoChiao, Yu-HsuanPantelakis, SpirosChiappone, AnnalisaPantelic, IvanaChiessi, EsterPanunzi, BarbaraChin, Alan H.Panyukov, SergeyChipara, MirceaPaolella, AndreaChiu, Chih-WeiPaolino, MarcoChiu, Fang-ChyouPapadopoulos, Antonios N.Chladek, GrzegorzPapagiannopoulos, AristeidisChmielarz, PawełPapagni, AntonioCho, ChungyeonPapoulis, DimitriosCho, Dong-WooParajó, Juan C.Cho, JunhanParajuli, DurgaCho, NamchulParale, Vinayak GCho, SunghunPáramo-García, UlisesChochos, ChristosParedes-Madrid, LeonelChoi, DongwhiParida, DambarudharChoi, Jae-HongParida, KaushikChoi, Seok KiParisi, FilippoChoi, Soo-HyungPark, Byung-DaeChoi, Sung-HwanPark, Chul-hoChortos, AlexPark, ChulhwanChou, Shih-FengPark, DaesungChow, James C. L.Park, Jang MinChowdhury, PankajPark, Jay HoonChrcanovic, BrunoPark, Ji-WonChrissafis, KonstantinosPark, Jong-SeokChristen, ThomasPark, JuhyunChristensen, Jørn BPark, Jun-BeomChristoforides, EliasPark, Keum HwanChruściel, Jerzy J.Park, Kyung MinChu, MiaoqiPark, MinChuang, Er-YuanPark, MiraChukov, DilyusPark, Seon JooChun, Heung JaeParkar, Shanthi G.Chung, Chan I.Parnian, Mohammad JavadChung, Neal Tai-ShungPasbakhsh, PooriaChung, Sheng-HengPascual-Seva, NúriaChung, Shyan-LungPashchenko, DmitryCiardelli, FrancescoPasinszki, TiborCicala, GianlucaPassaglia, ElisaCidade, Maria TeresaPastore, RobertoCielecka-Piontek, JudytaPaszkiewicz, SandraCieplak, MaciejPatel, JigneshkumarCiesielski, MichaelPatel, RajkumarCinelli, PatriziaPatelski, PiotrCiolaciu, DianaPatil, Swati J.Ciolacu, DianaPatra, NiranjanCipullo, RobertaPatrickios, C.Cirillo, GiuseppePattelli, LorenzoCoates, Nelson E.Patterson, JenniferCobos, AngelPăucean, AdrianaCochis, AndreaPaul, CharlesCodină, Georgiana GabrielaPaulsen, HaukeCoduri, MauroPavlič, MatjažCoelho, LuisPavlík, ZbyšekCohen Stuart, MartienPaz-Jiménez, EvaCoiai, SerenaPeak, Charles W.Cojocaru, CorneliuPeddapuram, AdithyaColin, XavierPedron, SaraColl, DeysmaPeer, PetraCollar, Emilia P.Pegoretti, AlessandroColom, XavierPei, ZhiqiangColtelli, Maria BeatricePeila, RobertaConcetta Valeria Lucia, GiosafattoPellicane, GiuseppeConesa, Juan A.Penava, DavorinConstantinescu, Catalin-DanielPenchev, HristoConstantinescu, Dan MihaiPeng, DengfengConte, ClaudiaPeng, Robert Y.Contescu, Cristian I.Peng, XinshengContessi Negrini, NicolaPenlidis, AlexanderCoons, J.Pepelnjak, TomazCoppola, BartolomeoPerale, GiuseppeCorcione, Carola EspositoPerche, FedericoCordeiro, Rosemeyre AmaralPercino, JudithCoronas Ceresuela, JoaquínPereira, Carlos DiasCorrea, Daniel S.Pereira, RuiCorreia, Sandra F. H.Pérez Puyana, Víctor ManuelCorrigan, NathanielPerez, ElenaCortaberria, GalderPerez-Alvarez, L.Cortés, Farid B.Pérez-Amodio, SoledadCortese, BarbaraPérez-Prieto, DanielCortez-Lemus, Norma AidéPerinelli, Diego RomanoCosco, DonatoPeriyasamy, Aravin PrinceCoseri, SergiuPerov, NikolayCosimbescu, LeliaPersson, IngmarCosta, PedroPerumal, SugunaCostabile, GabriellaPessoa, RodrigoCosta-Pinto, Ana RitaPessoa, Rodrigo SávioCoumes, FannyPester, ChristianCoutinho, Paulo José GomesPestov, AlexanderCovas, José AntónioPetcu, CristianCowan, Matthew GreigPeter, AncaCraciun, Eduard-MariusPeterson, Gregory I.Cran, Marlene J.Petr, ŠálekCrespi, StefanoPetrella, AndreaCretich, MarinaPetrič, MarkoCriado-Gonzalez, MiryamPetriccione, MilenaCrisan, OvidiuPétrissans, MathieuCrist, BuckleyPetronela, NechitaCristache, Corina MarilenaPetrou, PanagiotaCristea, DanPetrucci, RobertoCristea, Mihaela DanaPflaum, JensCristescu, RodicaPfleger, JiriCroitoru, CătălinPfnür, HerbertCroll, Andrew B.Pham, Chuyen VanCrucho, Carina Isabel CorreiaPham, Tien DucCruz, Juan C.Pham, TungCruz, SandraPhan, Hoang-PhuongCruz-Delgado, Víctor JavierPhilippe, AllanCruz-Villar, Carlos-AlbertoPhillips, JonathanCsapo, EditPiccirillo, ClaraCsoka, LeventePiedade, Ana PaulaCuan-Urquizo, EnriquePiegat, AgnieszkaCubero, DavidPielichowski, KrzysztofCui, HaitaoPierini, FilippoCui, ZhengPietropaolo, AdrianaCumming, Mathew HoaniPigossi, Suzane CristinaCure, JérémyPijls, BartCvek, MartinPilch-Pitera, BarbaraCyganowski, PiotrPilic, BrankaCysewska, KarolinaPilipović, AnaCzigány, TiborPilyugina, EkaterinaD’Adamo, IdianoPimanmas, AmornD’Amora, UgoPintauer, TomislavD’Amore, AlbertoPinto, DeesyD’Anna, FrancescaPinto, ErnaniD’hooge, Dagmar R.Piotrowski, TomaszDa Silva, Eduardo MoreiraPipiška, MartinDa’na, EnshirahPiras, Anna MariaDabos-Seignon, SylviePispas, StergiosDadashi-Silab, SajjadPistone, AlessandroDagmar, MerinskaPiszczyk, ŁukaszDai, BaiqianPitschmann, VladimirDai, HonglianPitto-Barry, AnaïsDai, HongqiPiwoński, IreneuszDai, LizongPizzi, AntonioDai, SongyuanPlatunov, MikhailDale, Erica A.Plevris, VagelisDalessandri, DomenicoPlikusiene, Ieva BaleviciuteDalle Vacche, SaraPlisko, Tatiana V.Dama, MuraliPobiega, KatarzynaDamodaran, KrishnanPodgórski, MaciejDan, DobrotaPodkoscielna, BeataDaniel, YosephPodkościelna, BeataDante, SilviaPogorielov, MaksymDao, Van-DuongPogrebnjak, AlexanderDargazany, RoozbehPoisel, HansDarie-Nita, Raluca NicoletaPoisot, Martha E.Darling, SethPolanski, MarekDaroch, MaurycyPoletto, MatheusDarwish, MohamedPolino, GiuseppinaDas, GautamPolitakos, NikolaosDas, Narayan ChandraPolitano, Grazia GiuseppinaDasari, AravindPoloskei, KornelDash, BirajaPolotskaya, GalinaDaugelavicius, RimantasPonomarev, Igor I.Davank, VadimPons, JosefinaDavarpanah, AfshinPontikoglou, CharalamposDave, SandeepPopa, LacramioaraDaví, FabrizioPopescu, AndreiDavies, PaulPopescu, Carmen-MihaelaDavis, FredPopescu, Vasile A.Dawn, ArnabPopescu, VasilicaDe Almeida, Jose ManuelPop-Georgievski, OgnenDe Araújo, Daniele RibeiroPopielarz, RomanDe Aza, Piedad N.Porcarelli, LucaDe Bonis, AngelaPosadas, PilarDe Caro, VivianaPospiech, DorisDe Cesare, FabrizioPotapov, AndreiDe Diego, AnaPourchet, SylvieDe Grooth, JorisPourrahimi, Amir MasoudDe Laporte, LauraPovolotskiy, Alexey V.De Leo, FilomenaPoy, GuilhemDe León, A. S.Prabakar, SujayDe Luca, PierantonioPrabakaran, MayakrishnanDe Marco, IolandaPrabhu, VivekDe Meijer, MariPradanos, PedroDe Nardo, LuigiPrajzler, VaclavDe Oliveira Barra, Guilherme MarizPramanik, ArindamDe Oliveira Lobo, AndersonPrat, MariaDe Oliveira, Helinando PequenoPrata, JoanaDe Paz, Antonio TaberneroPredoi, DanielaDe Santis, RobertoPresa Soto, M. AlejandroDe, RanjitPrhashanna, AmmuDeak, GyorgyPribyl, JuliaDębowski, Maciej ŁukaszPrice, GarethDeimede, ValadoulaProchazka, KarelDejam, MortezaProfire, LenutaDel Hoyo Martinez, CarmenProkopowicz, MagdalenaDelattre, CédricProto, AndreaDelattre, FrançoisPruncu, CatalinDelbecq, FrédéricPrytz, ØysteinDelgado, Luis M.Przybyłek, MaciejDelpouve, NicolasPrzybylek, PiotrDemers, VincentPtaszek, PawełDemey, HaryPucci, AndreaDemina, Tatiana S.Puchades, IvanDemir, BarisPuchalski, MichałDemir, MuslumPuchot, LauraDenchev, ZlatanPuglia, DeboraDeng, RenhuaPuglisi, RosariaDenizli, AdilPuiggalí, JordiDeryabina, Yu I.Pulidindi, Indra NeelDeterre, RémiPullano, Salvatore A.Dey, KamolPulyalina, AlexandraDhakshinamoorthy, AmarajothiPunta, CarloDhand, VivekPurcar, VioletaDhilip Kumar, Sathish SundarPustak, AnđelaDi Chenna, PabloPuszka, AndrzejDi Girolamo, RoccoPuttreddy, RakeshDi Liegro, ItaliaPyataev, Nikolay A.Di Maio, LucianoPycia, KarolinaDi Martino, AntonioPyo, SukhoonDi Noia, Luigi PioQaiss, AbouelkacemDi Palma, LucaQi, HuanDi Pierro, ProsperoQi, Yu-HongDias, Marcos LopesQian, ShaopingDíaz, David DíazQin, CaiqinDiaz, LilianaQin, YuyueDiaz, Sebastian AndresQin, ZhaoyeDíaz-Calderón, PauloQu, Jin-PingDiaz-Oltra, SantiagoQu, JinqingDiban, NazelyQu, XiaozhongDichiara, AnthonyQuan, DongDíez, EduardoQuang Trung, TranDijkstra, Bauke W.Quiles-Carrillo, LuisDikshit, VishweshQuirino, Rafael L.Dillon, Marina P. ArrietaR. El-Seedi, HeshamDimakopoulos, YannisR. Wellen, Renate MariaDing, JianxunRabbani, Mohammad MahbubDing, LiangRachtanapun, PornchaiDing, MingmingRadacsi, NorbertDing, Wei DanRadecka, IzabelaDing, ZhaoRadian, AdiDing, ZhaoyangRadtke, MariuszDinh, ToanRadu, EugenDinu, Maria ValentinaRadulescu, AurelDioguardi, MarioRaffa, PatrizioDiomede, FrancescaRaffaini, GiuseppinaDirè, SandraRafiee, MohammadDivins, Núria J.Raftopoulos, KonstantinosDixon, DavidRagauskas, ArthurDobosz, BernadetaRaghavan, ShreyaDobrotă, DanRaghunathan, VijayDobrzynski, PiotrRagusa, AndreaDodi, GianinaRahier, HubertDodiuk, HannaRahman, Md AnisurDoitrand, AurélienRahman, Mohammad MizanurDolganov, Pavel VladimirovichRahman, Mohammed M.Dolnik, BystrikRahme, KamilDołowy, MałgorzataRaimo, MariaDolzhenko, Anton V.Raimondo, MarialuigiaDomalik-Pyzik, PatrycjaRajan, RobinDomenici, ValentinaRajca, MariolaDominguez-Robles, JuanRajkhowa, RangamDominici, FrancoRal, Jean-PhilippeDong, LiangliangRamakumar, KinthadaDong, XufengRamamoorthy, AyyalusamyDorigato, AndreaRamamoorthy, Sunil KumarDorożyński, PrzemysławRamamoorthy, Sunil Kumar LindströmDörr, DominikRamanavicius, ArunasDos Reis, Paulo Nobre BalbisRamani, AlwarDos Santos, Arnaldo R.Ramaraj, SukanyaDos Santos, Demetrio JacksonRamtoola, ZebunnissaDos Santos, João Miguel MarquesRancan, MarzioDoustkhah Heragh, EsmaeilRanella, AnthiDrabowicz, JozefRangari, VijayDrache, MarcoRangelov, S.Dräger, GeraldRangou, SofiaDraghi, LorenzaRanzato, EliaDrissi-Habti, MonssefRao, AshitDrummer, DietmarRao, QiDu, ChunfangRapa, MariaDu, GuanbenRaposo, MariaDu, GuodongRapson, Trevor D.Du, HaishunRaquez, Jean-MarieDu, XinRasheed, TahirDu, XushengRassõlkin, AntonDu, ZhifengRastin, HadiDuan, XiaozhengRatnakumar, B. V.Duan, YongxinRaucci, Maria GraziaDubey, NileshkumarRaucci, UmbertoDudás, ZoltánRault, FrancoisDudek, GabrielaRavaioli, StefanoDudhipala, NarendarRaval, RasmitaDuhamel, JeanRavi Nair, JijeeshDulova, NiinaRavula, SudhirDulski, MateuszRayappan, John Bosco BalaguruDumas, LudovicRaza, FaisalDumee, LudovicRealinho, VeraDunderdale, Gary J.Reany, OferDunky, ManfredRebollar, EstherDuraccio, DonatellaRecek, NinaDurante, MassimoRecio, José ManuelDurazzo, AlessandraRedina, OlgaÐuretek, IvicaRedshaw, CarlDušková-Smrčková, MiroslavaRee, MoonhorDutkiewicz, MichałRefatul Islam, MohammadDutta, NabaReglero Ruiz, José AntonioDuvigneau, JoostRegnier, GillesDworak, AndrzejReifsnider, Kenneth L.Dymond, MarcusReinholds, IngarsDzida, MarzenaReinoso, Miguel Á.Dziurka, DorotaReinprecht, LadislavDzolic, ZoranReiter, GünterEbara, MitsuhiroRemes, Mgr. ZdenekEcker, MelanieRen, LiEconomopoulos, Solon P.Ren, PenggangEcorchard, PetraRen, YongEevers, WalterRenato E.F., BotoEgan, Paul FRendueles, ManuelEggenhöffner, RobertoRennhofer, HaraldEhm, ChristianResmini, MarinaEhrmann, AndreaRestivo, JoãoEjfler, JolantaReyes-López, Simón YobannyEkielski, AdamReza, Khan MamunEl Fray, MirosławaRezania, ShahabaldinEl-Aassar, MohamedŘezanka, MichalElakneswaran, YogarajahRial-Hermida, IsabelElango, JeevithanRibeiro, HelenaElder, RobertRiccardo, ScalengheElder, SteveRichert, MariaElena, Tiuc AncuţaRiess, GisbertElfalleh, WalidRigalli, Juan PabloElías-Zúñiga, AlexRijckaert, HannesElizalde, Luis E.Rimmer, StephenElkady, MarwaRinaudo, MargueriteElkaseer, AhmedRincón, Marina E.Elkoun, SaidRintoul, IgnacioEllinas, KosmasRissanou, Anastassia N.Elmas, SaitRitter, HelmutEl-Newehy, MohamedRivera-Armenta, José LuisElsaid, KhaledRizzarelli, PaolaElwakeel, KhalidRoberts, Aled DeakinEnachescu, M.Robertson, ChristopheEnesca, AlexandruRobles, EduardoEngelbrecht, RainerRobustillo Fuentes, Maria DoloresEnko, DietmarRocchigiani, LucaEnrione, JavierRochefort, Gael Y.Eremeyev, VictorRochfort, KeithEric, TillmanRocio-Bautista, PriscillaErukhimovich, IgorRodembusch, FabianoErwan, PaineauRodembusch, Fabiano S.Escalante, JaimeRodger, AlisonEscárcega Bobadilla, Martha VerónicaRodič, PeterEscorihuela, JorgeRodrigue, DenisEscudero, CarlosRodrigues, FranciscaEslami, HosseinRodrigues, JoãoEspinach, Francesc XavierRodriguez, Hortensia M.Espinosa, EduardoRodríguez, MarioEsquivel, KarenRodríguez, RafaelEsrafilzadeh, DornaRodriguez, Ruben J. SanchezEssawy, HishamRodríguez-Félix, Dora EveliaEstellé, PatriceRodríguez-Lara, Blas ManuelEstrany, FrancescRodríguez-Lorenzo, LuisEvangelopoulos, MichaelRogers, SimonEvans, Edward A.Rogers, Simon A.Evtugyn, GennadyRogulska, MagdalenaEwald, AndreaRoh, ChanghyunFabbri, PaolaRollini, ManuelaFabian, ZsoltRom, MonikaFagadar-Cosma, EugeniaRomagnoli, ManuelaFainberg, BorisRomán Guerrero, AngélicaFakhouri, Farayde MattaRomanov, D. A.Fakin, DarinkaRomero, Carlos SantiusteFalchetto, Augusto CannoneRonca, AlfredoFally, MartinRondeau-Gagné, SimonFan, JiantaoRonen, AvnerFan, JizhouRonga, DomenicoFan, Pui L.Roppolo, IgnazioFan, QinguoRossella, DoratiFan, WeizhengRossi, René MichelFan, ZhengjieRosu, DanFang, ChengRotaru, AndreiFang, LiangRousakis, TheodorosFang, Yi-PingRoy Choudhury, ShreyaFantoni, AlessandroRoy, SagarFarè, SilviaRoy, SayanFarina, IleniaRoy, SwarupFarjas, JordiRozga, PawelFarris, StefanoRozik, NehadFasquelle, ThomasRozwadowski, TomaszFatehi, PedramRozynek, ZbigniewFatimah, IsRozza, ArianeFatyeyeva, KaterynaRudawska, AnnaFedorchenko, AlexanderRudra, ArnabFei, TongRueda-Sánchez, Juan CarlosFeldblyum, Jeremy I.Ruggiero, AlessandroFelgueiras, HelenaRuiz De Ballesteros, OddaFeng, ChuangRuiz Rubio, LeireFeng, HongboRuiz-Rubio, LeireFeng, QichengRuseckaite, RoxanaFeng, XiamingRussell, Gregory T.Feng, YongjunRusso, FrancescaFerdous, WahidRusso, LuigiFérec, JulienRusso, MariaFerhan, Abdul RahimRusso, PietroFerji, KhalidRusso, TeresaFernandes, Carla Sofia GarciaRusu, DanielaFernandes, Célio Bruno PintoRusu, Laura CristinaFernandes, IsabelRusu, Radu-DanFernandes, Mariana Sofia PeixotoRutkowska, KatarzynaFernandes, Ricardo M. F.Rutkowska, MariaFernandez, CarlosRužiak, IvanFernandez, InmaculadaRybka, Jakub DaliborFernández, Juan PedroRydz, JoannaFernández-García, MartaRydzkowski, TomaszFernández-Gutiérrez, MarRykowska, IwonaFernandez-Lorente, GloriaRyszkowska, JoannaFernández-Romero, Juan ManuelRytlewski, PiotrFerrara, Maria AntoniettaRytöluoto, IlkkaFerraz, Emanuela PradoRyu Cho, Yu KyoungFerreira, FilipeRyu, SeongwooFerreira, GuilhermeRyu, Yu KyoungFerreira, Nuno R.Saakes, MichelFerreira, PaulaSabantina, LiliaFerreiro-González, MartaSabatini, ValentinaFerri, Jose M.Sabbah, MohammedFerri, José MiguelSabet, MaziyarFerruti, PaoloSabri, FirouzehFiedler, BodoSacchetti, AlessandroFiedot-Toboła, MartaSacco, PasqualeFievet, PatrickSachdeva, Ir. SumitFilimon, AncaSadmani, A.H.M. AnwarFilip, JaroslavSadovskaya, IrinaFilip, PetrSadowski, ŁukaszFilipecki, JacekSaeb, Mohammad RezaFilipova, IneseSaeedifar, MiladFilippov, Alexander P.Safaei, Mohammad RezaFiliz, VolkanSafian, RezaFini, PaolaSagnelli, DomenicoFiona, HattonSahay, RahulFiore, VincenzoSahoo, SumantaFiorelli, JulianoSaid Schicchi, DiegoFiori, StefanoSaielli, GiacomoFiorillo, LucaSain, SunandaFiorio, RudineiSaito, HiromuFischer, HolgerSaiz, FernanFlandin, LionelSajab, Mohd ShaifulFliedel, ChristopheSajdak, MarcinFlores Johnson, Emmanuel AlejandroSajkiewicz, PawełFlores, Mario E.Sakai, TadamotoFloudas, GeorgeSakthivel, Tamil SelvanFocaroli, StefanoSaladino, RaffaeleFolch-Mallol, Jorge L.Salamanca, Constain H.Fonseca-Alves, Carlos EduardoSalaoru, IuliaForaboschi, PaoloSalas-de La Cruz, DavidFord, Michael J.Sałasińska, KamilaFormela, KrzysztofSalaün, FabienFormosa-Dague, CécileSalca, Emilia-AdelaFortelný, IvanSalcedo, Walter J.Fracasso, GiulioSaleem, MuhammadFraddosio, AguinaldoSalehi, SarahFragoso, AlexSales, Rita De Cássia MendonçaFragouli, DespinaSalimon, AlexeyFrancesco, GalianoSalit, Mohd SapuanFranco, Camilo AndresŠalkus, TomasFranco, LourdesSallam, Hossam El-DinFrancolini, IolandaSalonen, LauraFranco-Urquiza, EdgarSalthammer, TungaFrankcombe, TerrySalvia, MichelleFranke, DanielaSalvio, RiccardoFrank-Kamenetskaya, Olga V.Samanta, SubarnaFraxedas, JordiSamperi, FilippoFreire, FaustoSánchez, ManuelFriák, MartinSánchez-Barba, Luis F.Friesenbichler, WalterSánchez-Vergara, María ElenaFrihart, CharlesSanchiz, JoaquinFrith, JessSandri, GiuseppinaFu, LiSandwall, PeterFu, LiyeSängerlaub, SvenFu, QiangSanjairaj, VijayavenkataramanFu, YuSanjust, EnricoFuchs, SabineSantamaría, Pedro AntonioFucikova, AnnaSantamaria-Echart, ArantzazuFuensanta, MonicaSantana, Marianella HernándezFuentes Pumarejo, Luis GuillermoSantiago-Hernández, HéctorFukuda, TakeshiSantini, AntonelloFuller, BryanSantoro, SergioFulvio, Pasquale FernandoSantos De Almeida, TâniaFuoco, AlessioSantos, Ronaldo G.Fuoco, TizianaSantos-Juanes, JorgeFurgal, JosephSantos-Ruiz, LeonorFurlani, FrancoSanz-Garcia, AndresFuso, FrancescoSapino, SimonaGaan, SabyasachiSapkota, JanakGabrić, DraganaSarafraz, Mohammad MohsenGabrielli, PaoloSarasini, FabrizioGaca, MagdalenaSaratale, Ganesh DattatrayaGahleitner, MarkusSarkar, Amit KumarGaidukovs, SergejsSasai, YasushiGain, Asit KumarSassani, AlirezaGaitzsch, JensSatoh, KotaroGalateanu, BiancaSatoh, ToshifumiGalembeck, FernandoSavkovic, BorislavGalic, KataSawpan, MoyeenuddinGalicia-Luna, Luis A.Sayed, MurtazaGalkin, MaximSayer, ClaudiaGallant, NathanSayers, Edward JGalli, GiancarloSbirrazzuoli, NicolasGallo, SimoneScaffaro, RobertoGama, Nuno V.Scala, AngelaGamez-Perez, JoseScalese, SilviaGan, LuScarfato, PaolaGan, Yong X.Scarso, AlessandroGanazzoli, FabioScattina, AlessandroGanesh Kumar, MeenashisundaramSchacher, FelixGanewatta, MitraSchadt, MartinGangarao, Hota V.Schaefer, JenniferGao, ChengdeSchartel, BernhardGao, ChunmeiScheril, GiuseppeGao, Jing-FengSchintke, SilviaGao, Jun-GuoSchiraldi, DavidGao, MingSchirhagl, RomanaGao, PeiyuanSchledjewski, R.Gao, YachenSchmid, ManfredGao, YanziSchmid, MarkusGao, YongfengSchmidt, BernhardGao, YueshengSchmidt, Bernhard V.K.J.Gao, YunkaiSchmidt, MichaelGarbovskiy, YuriySchnabel, ThomasGarcés, Josep L.Scholz, CarmenGarcía Payo, CarmenSchroeder, BobGarcia, Andres SanzSchroeder, CharlesGarcía, NuriaSchwaminger, SebastianGarcia-Deibe, AnaSchwarz, DanaGarcia-Garcia, LeonardoSchwarze, MichaelGarcia-Gonzales, CarlosSchwarz-Pfeiller, AnneGarcia-Granada, Andres-AmadorSchweizer, ThomasGarcía-Gutiérrez, Mari CruzSciarretta, FrancescaGarcía-Martínez, Jesús-MaríaScribante, AndreaGarcía-Martinez, Joaquín C.Scuracchio, CarlosGarcía-Peñas, AlbertoSedlacek, PetrGarcia-Salaberri, Pablo A.Sedlačík, MichalGargiulo, ValentinaSeelam, Prem KumarGarofalo, EmiliaSefat, FarshidGarozzo, DomenicoSeh, Zhi WeiGarrill, AshleySeide, GunnarGarrison, ThomasSekiguchi, YuGarvey, ChristopherSekkat, ZouheirGashti, Mazeyar ParvinzadehSelene, Sepulveda-GuzmanGautam, Siddharth S.Selin, VictorGautier Di Confiengo, G.Semaltianos, Nikolaos G.Gavrilov, NemanjaSemenyuk, PavelGazzano, MassimoSemino, RocioGe, Jun CongSemsarilar, MonaGe, QiSen Gupta, ArnabGe, TingSenel, MehmetGe, XinSeo, Young-SooGebauer, JanSerafim, LuísaGeorgakilas, AlexandrosȘerban, Bogdan CătălinGeorge, GejoSerenko, OlgaGeorge, GraemeSeri, PaoloGeorge, Sajan DanielSerpooshan, VahidGeorge, Thomas F.Serra, AngelsGeorgiev, VasilSerrano-Aroca, ÁngelGeorgopanos, ProkopiosSessini, ValentinaGerbaldi, ClaudioSeyyed Monfared Zanjani, JamalGericke, MartinSfarra, StefanoGeszke-Moritz, MałgorzataSgouros, AristotelisGeta, David A.Sgreccia, EmanuelaGhaani, MasoudSha, YeGhaffar, SeyedShafigh, PayamGhandi, KhashayarShah, Kwok WeiGhasemlou, MehranShah, ZahirGhica, Mihaela VioletaShaik, Mohammed RafiGholamipour-Shirazi, AzarmidokhtSham, Janet FCGholampour, AliakbarShamonin, MikhailGhonaim, HassanShan, XinGhori, Muhammad UsmanShangguan, YonggangGhosh, DebanjanaShankar, EswarGhosh, MonojShao, LuGhosh, ShramanaShapter, JoeGhosh, SubrataSharker, Shazid Md.Ghoshal, SushantaSharma, AnuragGiannakas, ArisSharma, BhavnaGibbons, Gregory JohnSharma, Piyush SindhuGierszewska, MagdalenaSharma, PriyankaGigante, VitoSharma, Priyanka R.Gigliotti, MarcoSharma, Sunil KumarGimeno Seco, MiquelSharopov, FarukhGindl-Altmutter, WolfgangShaulsky, EvyatarGioele, PagotShavandi, AminGiorcelli, MauroShayesteh Moghaddam, NargesGiorgini, LorisShcharbin, DzmitryGiraud, StephaneShe, Yong-XinGiri, GauravShehata, NaderGitsas, AntonisSheikh Mohamed, M.Gitsov, IvanShellaiah, MuthaiahGizdavic-Nikolaidis, MarijaShen, EricGkourmpis, ThomasShen, JialongGlavina, DomagojShen, JunGliszczynski, AdrianShen, KelvinGloria, AntonioShen, XiGlowinska, EwaShen, ZhiqiangGniewosz, MałgorzataSheng, BinGoda, IbrahimSheng, WenboGodina, RaduSheng, ZhanwuGodinho, Maria HelenaShevchenko, Valery V.Godiya, ChiragShi, AiminGoh, Guo DongShi, An-ChangGoh, KunliShi, JinshengGOH, Suat HongShi, KaiyuanGohari, SoheilShi, QiangGok, OzgulShi, SheldonGola, DeepakShi, SiqiGolewski, PrzemysławShi, TongfeiGolub, IgorShi, YiGomes, Ana P.Shi, YongqianGomes, HenriqueShi, Yu-GangGomes, Henrique LeonelShi, YushengGomes, Joao FShibaev, PetrGomez Cerezo, Maria NatividadShie, Ming-YouGomez, David GarozShikov, AlexanderGómez, XabierShimomura, TakeshiGómez-Soberón, Jose ManuelShin, Eun JooGomha, SobhiShinde, SudhirkumarGonçalves, M. Sameiro T.Shinde, SurendraGonçalves, RenatoShinde, Surendra KrushnaGong, XiongShinya, AkikazuGonzalez Nunez, RubénShirahata, NaotoGonzález, AlbaShirinbayan, MohammadaliGonzalez, Jimena S.Shishkin, AndreiGonzález, JoseShoda, MakotoGonzález, SergioShrestha, KshitijGonzález-Benito, JavierShtenberg, GiorgiGonzalez-Gutierrez, JoaminShuai, WangGonzález-Marcos, María P.Siamidi, AngelikiGonzález-Núñez, RubénSideratou, ZiliGonzato, CarloSidhureddy, BoopathiGooneie, AliSidorenko, AlexanderGopalan, PadmaSigolaeva, Larisa V.Gopalan, SaianandSikora, JanuszGorgieva, SelestinaSilantyeva, Elena A.Gorji, Nima E.Siller, HectorGórny, KrzysztofSilva Filho, EdsonGościańska, JoannaSilva, JoãoGoseki, RaitaSilva, JorgeGossage, R. A.Silva, Tiago AlmeidaGossetti, FrancescoSilveira, Nádya P. DaGotzias, AnastasiosSilvestri, MarcoGourinat, YvesSimari, CataldoGouveia, Rubia FigueredoSimon, ValérieGoyanes, Silvia NairSimončič, BarbaraGradov, Oleg V.Simorangkir, Roy B. V. B.Grande Tovar, Carlos DavidSimula, AlexandreGrassi, MarioSing, Swee LeongGrätz, SvenSingh, Ajay VikramGrause, GuidoSingh, GautamGreco, AntonioSingh, InderbirGregori, AlbertoSingh, JitendraGriffini, GianmarcoSingh, Jitendra KumarGrijalvo, SantiagoSingh, RajbirGrochowicz, MartaSingh, RajeshGroza, AndreeaSingha, ShuvraGrumezescu, Alexandru MihaiSingha, SubhankarGrunlan, Jaime C.Sinha Ray, SaikatGryszel, MaciejSinha Ray, SumitGryta, MarekSinha, JasmineGu, ChunjuSinha-Ray, SumanGu, HongboSinjari, BrunaGu, JunweiSionkowska, AlinaGu, QilinSiqueira, NatalyGu, YunqingSirvio, JuhoGuan, QingbaoSivaramapanicker, SreejithGuangjiu, ZhaoSixta, HerbertGuedes, Rui MirandaSkórczewska, KatarzynaGuedes-Alonso, RaycoSkorik, Yury A.Güemes, AlfredoSkrifvars, MikaelGuerra-Sanchez, GuadalupeSkupov, Kirill M.Guerrero-Vaca, GuillermoSkvortsov, Ivan Yu.Guerrieri, NicolettaSlomkowski, StanislawGui, RijunSłomkowski, StanislawGui, ZheSlouf, MiroslavGuidoin, RobertSlouka, ZdenekGuijun, XianSmardzewski, JerzyGulicovski, JelenaSmith Callahan, Laura A.Gumfekar, SarangSmith, Adam E.Gun’ko, Vladimir M.Smith, Maree T.Gunde, Marta KlanjsekSmith, Paul F.Gunia-Krzyżak, AgnieszkaSmolka, PetrGunji, TakahiroSnežana, PapovićGuo, BaolinSoares, JulioGuo, BinSobkowicz, Margaret J.Guo, DandanSobol, Emil N.Guo, JiangSocol, MarcelaGuo, WeihongSodano, FedericaGuo, Zhan-HuSokolova, Maria P.Guo, Zhao-XiaSokolova, Maria PetrovnaGupta, AnjuSola, DanielGupta, ArvindSolano, FranciscoGupta, CharuSolovieva, AnastasiyaGupta, MohitSomberg, JulianGupta, SanjuSong, BoGupta, SiddharthSong, HengxuGurikov, PavelSong, JianweiGushchin, IvanSong, JinshengGuskova, OlgaSong, JuhaGutierrez Ramirez, ManuelSong, JunlongGutierrez, JunkalSong, KenanGutt, GheorgheSonnier, RodolpheGuzik, MaciejSopiha, Kostiantyn V.Guzmán, EduardoSorrentino, AndreaGuzman, SusanaSorrentino, LuigiGyarmati, BenjáminSoto, FernandoGyörgy, KaszaSotta, PaulGzara, LassaadSouza, Victor LaurianoHagita, KatsumiSpagnoli, AndreaHahn, Jae RyangŠpanić, NikolaHaider, JulfikarSparks, ConradHaladjova, EmiSpasojevic, PavleHamouda M, MousaSpasova, MariyaHampton, JenniferSperanza, VitoHan, DaehoonSpina, RobertoHan, DehuiSpinelli, GiovanniHan, Dong-WookSpinelli, LucianaHan, JingquanSprenger, StephanHan, YingchaoSprynskyy, MyroslavHan, YousooSriram, GopuHanafy, Nemany A. N.Sriroth, KlanarongHandge, Ulrich A.Stachewicz, UrszulaHandral, HarishStadler, FlorianHanieh, Patrizia NadiaStaicu, TeodoraHanif, AsadStana, Anca-DanielaHaniu, HisaoStancu, Izabela-CristinaHansma, HelenStankova, NadyaHanyková, LenkaStapleton, Scott EdwardHao, NanjingStaszak, KatarzynaHao, XiaojuanStavropoulos, Sotirios G.Haponiuk, JozefStefanelli, ManuelaHaque, RezwanulStejskal, JaroslavHara, MasanoriStelescu, Maria DanielaHaraźna, KatarzynaStelzer, FranzHardy, John G.Stenzel, VolkmarHardy, John GeorgeStepanov, Gennady V.Harja, MariaSterzyński, TomaszHarmandaris, VagelisSteup, MartinHarmandaris, Vagelis A.Stobiecka, MagdalenaHarrisson, SimonStochino, FlavioHart, KyleStöckelhuber, Klaus WernerHartings, Matthew R.Stogniy, Marina Yu.Hasan, AbsharSTOIĆ, AntunHasanzadeh Kafshgari, MortezaStoica, AnicutaHasegawa, MakotoStoikov, Ivan IvanovichHashidzume, AkihitoStojanović, SanjaHashimi, SaeedStojmenović, MarijaHashimoto, MasanoriStokke, Bjørn TorgerHassan, Mohammad K.Stolarczyk, AgnieszkaHassanin, HanyStoleru, ElenaHatoum, HodaStolojan, VladHatzikiriakos, Savvas G.Stommel, MarkusHaupt, MichaelStoppelkamp, SandraHaviarova, EvaStorti, GiuseppeHayashi, KeiStoyan, DietrichHayashi, TakuyaStoychev, GeorgiHayes, Simon A.Strąkowska, AnnaHe, GuanjieStrankowska, JustynaHe, HongliangStride, John ArronHe, JianxinStrumia, Miriam CristinaHe, ShaojianStrunskus, ThomasHe, Wei-DongStruszczyk, Marcin HenrykHe, WeilueStrzelec, KrzysztofHe, YongStrzemiecka, BeataHeijungs, ReinoutStumpe, JoachimHeinrich, BenoîtStuparu, MihaielaHejna, AleksanderSturgis, JamesHeljak, MarcinSturm, HeinzHellmich, UteStylianakis, MinasHellweg, ThomasSu, An-ChungHelsch, GundulaSu, Chen-YingHenkel, CarstenSu, ZhouchengHerbert, David E.Subbiahdoss, GuruprakashHernández-Cordero, JuanSubramanian, SundarrajanHernandez-Martinez, Angel R.Sudesh, KumarHernández-Núñez, EmanuelSudo, AtsushiHernandez-Vazquez, Jesus-MariaSuen, Maw-CherngHerrera-Franco, Pedro JesúsSugahara, TakeshiHerrera-May, Agustin L.Sugawa, KosukeHerrera-Ordonez, JorgeSugiyasu, KazunoriHerres-Pawlis, SonjaSukhorukov, GlebHeß, MarkusSulaeva, IrinaHess-Dunning, AllisonSulaiman, YusranHevus, IvanSulka, Grzegorz DariuszHidalgo Salazar, Miguel ÁngelSumelka, WojciechHidalgo, JavierSummerscales, JohnHiejima, YusukeSun, FaqianHilliou, LoicSun, HaoHinman, SamuelSun, JiazhenHirai, TomoyasuSun, JingyaoHirayama, FumitoshiSun, LuHizal, GürkanSun, MengHiziroglu, SalimSun, ShaolongHo, Chia-cheSun, TaoHo, Ko-ShanSun, XiuxuanHoang, Van-ThoSun, ZhiqiangHofmann, AndreasSunayama, HirobumiHofmann, ElisabethSundar, Victor JohnHolzer, ClemensSundarrajan, SubramanianHomaeigohar, ShahinSundberg, AnnaHong, Jung-IlSung, Wen-ChiehHong, KunlunSuplicz, AndrásHong, LiangSurace, RossellaHong, RuoyuSuraneni, PrannoyHong, Sung-KyuSurmenev, Roman A.Hong, TaoSu-Wen, HsuHönicka, MarkusSuzuki, NozomuHonzíček, JanSvetlana, Tretsiakova-McNallyHorrocks, A. RichardSvoboda, JanHorsky, JiřiSweeney, JohnHortigüela, María J.Świderski, WaldemarHorváth, JuditSwinarew, AndrzejHossain, M. NurSysel, PetrHossain, MokarramSzałowski, KarolHossain, MuhammadSzczepanski, Caroline R.Hosseini Nejad, ElhamSzekely, GyorgyHosseinkhani, HosseinSzewczyk, RomanHou, JingweiSzilágyi, IstvánHou, LinxiSzkoda, MariuszHou, YongSznitowska, MałgorzataHou, ZhaoshengSzostak, ElżbietaHowell, BobSzostak, MarekHoy, RobertSzubert, KarolHsiao, Hsi LienSzukalski, AdamHsiao, Yu-ShengSzymborski, TomaszHsissou, RachidSzymczyk, AnnaHu, AnmingTabacaru, AurelHu, Ching-YaoTabata, YasuhikoHu, JinlianTabish, TanveerHu, JinmingTadiello, LucianoHu, JiyongTadyszak, KrzysztofHu, LiangTae-Jun, HaHu, Shang-HsiuTahara, HironobuHu, XianglongTaheri, ShimaHu, YangTaheri-Qazvini, NaderHu, YujinTakafuji, MakotoHuang, BoyangTakahashi, KazuakiHuang, CaoxingTakahashi, KazuoHuang, Chih-FengTakahashi, ShuheiHuang, Chi-HsienTakamuku, ShogoHuang, Chun-YungTakaya, TomohisaHuang, GuoshengTakayanagi, ToshiyukiHuang, Haw-MingTakayoshi, SuzukiHuang, HeTakenaka, MikihitoHuang, JianyingTakeoka, ShinjiHuang, JinTakeshita, SatoruHuang, KunTalebian, SepehrHuang, Ming-ShyanTalò, GiuseppeHuang, QijinTam, ChristinaHuang, QiyaoTamai, ToshiyukiHuang, Shuan-YuTamaru, YutakaHuang, WeiminTame, JeremyHuang, YifengTamm, TarmoHuang, YuchengTampieri, FrancescoHuang, YugangTamura, AtsushiHuang, ZhaohuiTamura, HrioshiHuerta-Angeles, GloriaTan, ChenHuling, JenniferTan, DanielHumar, MihaTan, Jeremy P. K.Humelnicu, DoinaTan, QinggangHunt, Christopher G.Tan, WeiHuntosova, VeronikaTan, YebangHussain, BabarTanaka, TomonariHussin, Mohd. HazwanTanase, CorneliuHutmacher, DietmarTańczyk, MarekHuynen, IsabelleTang, ChaolongHuynh, Thanh-CanhTang, LongtengHuyop, Fahrul ZamanTang, NaipengHwang, Cheol-HongTang, Ren-ChengHwang, Gi-ByoungTang, YouhongHwang, Sheng-JyeTang, YunlongHwang, SungyeonTangadanchu, Vijai Kumar ReddyHyun, KyuTaniguchi, TsuyoshiIanchis, RalucaTański, TomaszIandolo, DonataTao, FeifeiIatsunskyi, IgorTao, KaiIbarz, ElenaTappa, KarthikIbrahim, Sherif AbdelazizTarabanko, Valery E.Ibrahim, ToniTarbuk, AnitaIconaru, Simona LilianaTarrés, QuimIdris, Maizlinda IzwanaTatullo, MarcoIezzi, GiovannaTauber, Uwe CIfelebuegu, AugustineTaubert, AndreasIghigeanu, DanielTaylor, Alan G.Iglesias-Martínez, EmiliaTcherdyntsev, Victor V.Ignés-Mullol, JordiTecpoyotl-Torres, MargaritaIkehara, KenjiTefferi, MattewosIl’ina, Marina V.Teixeira, Marcos F.S.Ilg, PatrickTellez Jurado, LuciaIlia, GheorgheTeng, PengIlic-Stojanovic, SnezanaTenhaeff, WyattIliescu, CiprianTeodorescu, FlorinaIllescas, JavierTeramoto, HidetoshiInagaki, NorihiroTeramoto, NaozumiInomata, KatsuhiroTerao, KenIoannides, TheophilosTercjak, AgnieszkaIoelovich, MichaelTerekhova, Irina V.Ion, Rodica-MarianaTerzopoulou, ZoeIordanskii, Alexey L.Terzopoulou, ZoiIorizzo, MassimoTestani, ClaudioIpsakis, DimitrisThakur, VijayIqbal, Hafiz M. N.Thakur, Vijay KumarIrie, MasaoThang, Tran QuyetIrshad, KashifThapa, Raj KumarIsaeva, Vera I.Theato, PatrickIsakov, DmitryTheres Baby, TessyIshibashi, JacobThi, Thai Thanh HoangIshida, YuichiThitsartarn, WarintornIslam, MohammadThomas, NoelIslamoglu, TimurThompson, Richard L.Ismail Abu-Jdayil, Basim MohammadThompson, RoneyIsmail Mourad, Abdel-HamidThoniyot, PraveenIsola, GaetanoThrivikraman, GreeshmaIsono, TakuyaTian, WeiguoIsreb, AbdullahTiemblo Magro, PilarIssi, Jean-PaulTiera, MarcioIta, KevinTietze, RainerIuco Castro Da Silva, IgorTijing, LeonardIván, BélaTikhonov, VladimirIvančević, DarkoTimashev, PeterIvanets, AndreiTimon, RabczukIvanišová, EvaTing, Jeffrey M.Ivannikov, A. Yu.Titomanlio, GiuseppeIvchenko, PavelTjong, Sie ChinIwamoto, TakeshiTodaro, LuigiIwan, AgnieszkaTodea, AnamariaIzumi, YudaiTohmura, Shin-ichiroJabbari, MasoudToikka, AlexanderJabbarzadeh, AhmadTokarska, MagdalenaJabraoui, HichamTokuda, GakuJacquel, NicolasTokuyama, HideakiJadidi, MehdiToledano-Osorio, ManuelJafarinejad, ShahryarTolkou, AthanasiaJahandar Lashaki, MasoudTolstoy, Peter M.Jain, GauravTomaszewska, EwaJakóbik-Kolon, AgataTomczyk, KrzysztofJakubczak, PatrykTomić, SimonidaJamalabadi, Mohammad Yaghoub AbdollahzadehTomita, YasuoJammalamadaka, UdayabhanuTomovska, RadmilaJamrógiewicz, MarzenaToncheva-Moncheva, NataliaJamróz, PiotrTondi, GianlucaJamshidi, AliTonelli, Alan EdwardJanarthanan, GopinathanToprakci, Hatice Aylin KarahanJanas, DawidTopuz, FuatJanczarek, MonikaTorgal, F. PachecoJanda, ElzbietaTornabene, FrancescoJaneta, MateuszTörök, TamásJang, Keon-SooToroń, BartłomiejJankauskaite, VirginijaTorres Marques, AntonioJanossy, IstvanTorres Vaamonde, José EnriqueJanoušková, OlgaTorres-Giner, SergioJanovák, LászlóTorres-Lagares, DanielJansen, JohannesTorres-Ramírez, EduardoJanuszewski, RafałTosello, GuidoJanuszko, AdamTǒth-Katona, TiborJarosz, TomaszTrache, DjalalJarząbek, BożenaTrcek, JanjaJarzebski, MaciejTrček, JanjaJasinski, Jaroslaw JanTreviño, EstherJasiurkowska-Delaporte, MałgorzataTrimaille, ThomasJauregui, CatherineTrindade, AnaJeerapan, ItthiponTrivedi, VivekJelčić, ŽelimirTrocino, StefanoJelonek, KatarzynaTroiani, EnricoJena, Himanshu SekharTronci, GiuseppeJenny, Titus A.Trotta, FrancescoJeon, Jong-RokTrovato, FabioJeon, Ju-WonTrujillo-Cayado, LuisJeon, Young-PyoTruong, Nghia P.Jeong, Heon-HoTrzaskowska, MonikaJeong, SeriTrzebicka, BarbaraJeong, YoungjinTsai, Ang-ChenJerabek, MichaelTsai, Chia-Hung DylanJerca, AdrianaTscheliessing, RupertJerca, Florica AdrianaTsehaye, Misgina TilahunJerca, ValentinTseng, Hui-hsinJesionek, MarcinTsibidis, GeorgeJesionowski, TeofilTsinopoulos, IoannisJeziorska, ReginaTsoi, James Kit-honJha, HaritTsuji, HidetoJho, Eun HeaTsukegi, TakayukiJi, JunhuiTsurkan, Mikhail V.Ji, XianglingTsutsui, MakusuJi, Yan-LiTsvetanov, ChristoJia, BaoTu, GuoliJia, XudongTu, Ting-YuanJia, ZhongfanTu, ZhengkaiJian, RongkunTudor, Eugenia MarianaJian, XigaoTufa, Ramato AshuJiang, BinTumarkin, AndreyJiang, BingyanTunesi, MartaJiang, ChengTung, Shih-HuangJiang, ChengminTurczyn, RomanJiang, DegangTurner, DavidJiang, GuohuaTurner, Matthew S.Jiang, HongchuanTurng, Lih-ShengJiang, JiahuanTyliszczak, BożenaJiang, JunTzounis, LazarosJiang, KinorUdal’tsov, AlexanderJiang, LaiUdalov, OlegJiang, LinUddin, Sardar M. ZiaJiang, ShaohuaUde, AlbertJiang, WeiUeda, MotokiJiang, XueliangUetani, KojiroJiang, ZaixingUgarte, LorenaJiang, ZhihuaUjhelyiova, AnnaJiang, ZiwenUlasov, IlyaJiao, XuechenUlewicz, MalgorzataJiao, YuboUline, Mark J.Jin, MingUllah, Muhammad WajidJin, YifeiUlven, Chad A.Jin, ZhongminUmer, RehanJindřich, JindřichUmerska, AnitaJing, HuiUnnithan, Ranjith RajasekharanJoão, SilvaUppulury, KarthikJohannes, Hans-HermannUrakawa, OsamuJohanson, UrmasUshakov, Nikolay M.Johansson, ChristerUzunlar, ErdalJohnson, Boris GabrielV. Likhanova, NatalyaJohnston, Linda J.Vadahanambi, SridharJones, JustinVagin, MikhailJoseph, J. MarcinkoVahabi, HenriJoshi-Navare, KasturiVaitkus, SauliusJózsef Gábor, KovácsValente, MarcoJucius, DaliusValera, Ticiane SantosJuhasz, KataValero, Manuel FJulian, FernandoVallejo-Giraldo, CatalinaJulian-Lopez, BeatrizValvo, Paolo S.Jun, ByungMoonVamanu, EmanuelJun, Yun-SeokVan Nhien, Albert NguyenJunaidi, Mohd Usman MohdVan Rijn, PatrickJung, Hyun-DoVan Steenberge, Paul Hm M.Jung, Jae HwanVana, PhilippJung, JaehanVanangamudi, AnbharasiJung, JinmuVanderzande, DirkJung, SeunhoVannozzi, LorenzoJung, Yong ChaeVaquero, MonicaJungst, TomaszVaradwaj, PradeepJuodkazis, SauliusVaraprasad, KJůza, JosefVardhan, HarshK. Sharma, SunilVarganici, Cristian-DragosKaczmarska, KarolinaVargas Jentzsch, PaulKadam, AvinashVarley, RussellKadokawa, Jun-ichiVarma, Vijaykumar B.Kaehr, BryanVarol, H. SametKafarski, PawelVarvaresou, AthanasiaKairytė, AgnėVasiljević, JelenaKalali, Ehsan NaderiVassiliadis, SavvasKalantar-zadeh, KouroshVatani-Oskouei, AsgharKale, ManojVaudreuil, SebastienKaliappan, SenthilVaughan, AlunKaliva, MariaVázquez, José AntonioKallitsis, JoannisVázquez-Ovando, AlfredoKamal, Muhammad ShahzadVega, AlejandroKambhampati, Siva PramodhVekas, LadislauKaminaka, HironoriVelázquez Blázquez, José SebastiánKaminski, KamilVelázquez, RamiroKamiński, MarcinVélez-Cordero, Juan RodrigoKaminski, TomaszVenanzi, MarianoKammakakam, IrshadVenditti, IoleKamps, Jan HenkVendittozzi, CristianKan, Chi-waiVener, Mikhail V.Kananovich, Dzmitry G.Venkataraman, ShrinivasKanbayashi, ToruVenkatesan, JayachandranKandelbauer, AndreasVenugopal, DhamodharanKandemir, AliVenuti, ValentinaKandhasamy, SathiyarajVerduzco, RafaelKanematsu, HideyukiVergara, DiegoKang, Byung-jaeVerna, AlessioKang, HyeonggonVernardou, DimitraKang, HyoVeronesi, FedericoKang, MinjeeVerros, GeorgeKang, Sang SunVerzak, ŽeljkoKang, Sang WookVesel, AlenkaKang, Sung MinVia, BrianKang, WanliVidakis, NectariosKao, Chai-LinVidal, FernandoKapcia, Konrad JerzyVidic, JasminaKarakas, SertacVignali, AdrianoKarakoç, AlpVijayaraghavan, VenkateshKarakurt, IlbeyVikulina, AnnaKaraseov, PlatonVila, CarlosKaravasili, ChristinaVilaplana, FranciscoKarayiannis, NikosVilas, Jose LuisKarcz, DariuszVilaseca, FabiolaKareiva, AivarasVilla, CristianKargarzadeh, HaniehVillalobos, Luis FranciscoKarimov, DenisVillani, VincenzoKarimzadeharani, AtefehVillanueva-Llauradó, PaulaKarnati, Konda ReddyVillarreal-Gómez, Luis JesúsKarpacheva, G. P.Villaverde, GonzaloKarpiński, Tomasz M.Villone, Massimiliano MariaKaruppan, Mohan Kumar MuthuViloria Sáez, PaolaKaruppasamy, K.Vilsinski, Bruno HenriqueKaseem, MosabViña, JaimeKashaninejad, NavidVinothkannan, MohanrajKashi, SimaVirkki, MattiKashif, MuhammadViskadourakis, ZachariasKasmi, NejibVitale, AlessandraKasnatscheew, JohannesViuda-Martos, ManuelKasoju, NareshVivaldo-Lima, EduardoKatogi, HideakiVivet, AlexandreKatunin, AndrzejVlachopoulos, JohnKaushik, Nagendra KumarVlad-Bubulac, TachitaKawasaki, HideyaVlase, SorinKays, DeborahVodnar, Dan C.Kazmi, Syed Minhaj SaleemVögele, MartinKazuhiro, ShikinakaVogiatzis, GeorgiosKe, YangchuanVoicu, RodicaKean, ThomasVoicu, Stefan IoanKee, ScholtenVoliani, ValerioKehr, Nermin SedaVolker, KörstgensKeith, AttenboroughVolkov, VladimirKéki, SándorVordos, NickKelarakis, AntoniosVorndran, ElkeKeller, AdrianVoronov, AndriyKengelbach-Weigand, AnnikaVouyiouka, StamatinaKenry, K.Voyiatzis, GeorgeKepinska, MartaVoznyak, YuriKeplinger, TobiasVozzi, GiovanniKerch, GarryVrana, Nihal EnginKesharwani, Siddharth S.Vrsaljko, DomagojKeshk, SherifVu, Cuong ManhKhabaz, FardinVuluga, ZinaKhachatryan, GoharVyazovkin, SergeyKhalf, AbdurizzaghWach, RadosławKhalid, HammadWagenknecht, UdoKhan, AnzarWajda, AleksandraKhan, FasihullahWakioka, MasayukiKhan, Foysal ZahidWalach, WojciechKhan, ImranWałęsa-Chorab, MonikaKhan, Mohammad EhtishamWan, ChaoyingKhan, Omar F.Wan, Terng-JouKhandaker, MorshedWang, AiqinKharisov, Boris I.Wang, BaominKhattab, TawfikWang, BoKhlestkin, VadimWang, CaiKhmelev, Vladimir N.Wang, ChangxiangKhodadadian, AmirrezaWang, ChenguoKhoshnood, AbbasWang, Chih-FengKhosravani, Mohammad RezaWang, Chih-KuangKhotimchenko, Maxim Y.Wang, ChunhuaKhudyakov, Igor VWang, FangKhurshid, ZohaibWang, FeiKiefer, RudolfWang, FeipengKierys, AgnieszkaWang, FengKikionis, StefanosWang, Fu-MingKim, Ae RhanWang, FuzhouKim, B.K.Wang, HaopengKim, BongjuWang, HongxiaKim, ChoongikWang, HuKim, FelixWang, HualinKim, Gue-HyunWang, HuiKim, Hae-WonWang, JeffreyKim, Hyo JungWang, Jian-WenKim, HyunWang, JinchengKim, Hyun ChanWang, JingKim, HyungsupWang, JinyanKim, HyunilWang, JunKim, Hyun-JoongWang, JunjieKim, Jae YoungWang, JunshengKim, JaewooWang, KunKim, JooheonWang, LeiKim, JooyounWang, LifengKim, JuhyungWang, LingKim, Jung J.Wang, LiquanKim, Jung KyuWang, NaKim, JunghyunWang, NannanKim, Jun-HyunWang, PaiKim, Kyung HoWang, PengKim, Kyung-MinWang, PengfeiKim, Mi-JeongWang, Peng-YuanKim, Min-JungWang, Ping-liKim, MinkookWang, QiguiKim, MyungwoongWang, QingQingKim, Nam KyeunWang, QingsongKim, Pyung-HwanWang, QinyiKim, SangwonWang, RenxinKim, SeokminWang, RuibinKim, Seong YunWang, RuixueKim, SeungjuWang, San-LangKim, SokWang, ShaokaiKim, SunghanWang, Sheng-ShihKim, Sung-HoonWang, ShifengKim, TaehoonWang, ShigeKim, Tae-hwanWang, ShixingKim, Tae-HyunWang, TaoKim, WonhoWang, TonghuaKimura, YoshiharuWang, WeiKinashi, KenjiWang, WeiguangKireev, DmitryWang, WeihongKirker, GrantWang, WenshengKiselev, Konstantin V.WANG, XiaoenKishore, VidyaWang, XiaofengKiss, ImreWang, Xiaoguang (William)Kitahata, HiroyukiWang, XiaohongKitazawa, TakafumiWang, XilinKlar, AgnesWang, XinKlebert, SzilviaWang, XiutongKletschkowski, ThomasWang, XuechuanKlonos, PanagiotisWang, XunchangKlyui, Nickolai I.Wang, YachaoKo, Jang MyounWang, YanbinKo, Jiunn- LiangWang, YanleiKo, SeunghyunWang, Yen-ZenKobayashi, JunWang, YingKocherbitov, VitalyWang, YiningKoibuchi, HiroshiWang, YixiangKoikawa, MasayukiWang, YuKoivisto, JanneWang, Yun-CheKojima, ToshikatsuWang, YunmingKolesińka, BeataWang, ZhaoKoller, MartinWang, ZhijianKolniak-Ostek, JoannaWang, ZhiliangKolya, HaradhanWang, ZhiweiKolzunova, LidiiaWang, ZhongpengKomarov, Pavel V.Wang, ZhongyangKomives, TamasWang, ZongliangKondo, HiromuWang, ZongqianKong, Ling BingWan-Wendner, RomanKong, XianWatanabe, WataruKong, XiangruiWatts, John FKonieczkowska, JolantaWawrzkiewicz, MonikaKonnerth, JohannesWcisło, AnnaKontargyri, VassilikiWeber, ChristineKontziampasis, DimitrioWeber, DanielKontziampasis, DimitriosWeber, MartinKopel, PavelWebster, JohnKormaníková, EvaWeems, Andrew C.Korniejenko, KingaWei, GangKorolkov, Ilya V.Wei, HuaKorzeniewska, EwaWei, JiaKorzhikova-Vlakh, EvgeniaWei, JunchaoKorznikova, ElenaWei, QiangKoseva, NeliWei, RenboKoslowski, Hans RudolfWei, ZhaojunKosmulski, MarekWei, ZhongbaoKostić, Aleksandar Ž.Wei, ZhuKostjuk, SergeiWeis, MartinKostka, LiborWeiss, Clemens K.Kostrzewa, MarcinWen, JianmingKotsilkova, RumianaWen, YimeiKotsiomiti, EleniWen, ZhiweiKotula, AnthonyWeng, CanKotz, FrederikWeng, ZhihuanKoumoulos, Elias P.Wertz, Philip W.Koutahzadeh, NeginWess, TimKoutný, MarekWhittington, StuartKovačević, StanaWibowo, DavidKovalchuk, OleksandrWie, Jeong JaeKovalchuk, VolodjaWieckiewicz, MieszkoKovalcik, AdrianaWieczorek, Władysław G.Kowalczewski, PrzemysławWiedman, GregoryKowalczewski, Przemysław ŁukaszWieland, D. C. FlorianKowalczuk, MarekWieleba, WojciechKowalczyk, TomaszWiessner, SvenKowalewska, AnnaWilczyński, KrzysztofKowalski, GregoryWillberg-Keyriläinen, PiaKowaluk, GrzegorzWillenbacher, NorbertKowtharapu, Bhavani S.Willert, AndreasKoyama, YasuhitoWilpiszewska, KatarzynaKozakiewicz, JanuszWinczek, JerzyKozanecki, MarcinWinkler, KrzysztofKoziol, MateuszWinter, Horst HenningKozior, TomaszWiosna-Sałyga, GabrielaKozlowska, JustynaWiraja, ChristianKozłowska, JustynaWisian-Neilson, PattyKragović, MilanWitkowski, ArturKramarenko, Elena YuWłoch, MarcinKraśniewska, KarolinaWojtunik-Kulesza, Karolina AnnaKratochvilova, IrenaWolski, KarolKravchenko, Oleksandr G.Wong, Him ChengKremer, FriedrichWong, Leong SingKressler, JörgWong, Michael YinKristak, LubosWong, Roong JienKristl, MatjažWong, WingKritikos, GeorgiosWoodward, Robert T.Kritis, AristeidisWorley, S.D.Krivenko, PavelWorzakowska, MartaKröger, MartinWoszuk, AgnieszkaKról-Kilińska, ŻanetaWrona, MagdalenaKronekova, ZuzanaWu, BingKrumpfer, JosephWu, GuangleiKrystofiak, TomaszWu, Gwo-MeiKuang, QinWu, HongjingKuang, TairongWu, HongwuKuang, XiaoWu, HuaKubasov, IlyaWu, Hung-ChinKubiak, KatarzynaWu, Jing-YunKubo, MasatakaWu, JosephKuciel, StanisławWu, KangningKucinska-Lipka, JustynaWu, LiliKucińska-Lipka, JustynaWu, MinKucinski, KrzysztofWu, Shin-TsonKuckling, DirkWu, SiKudaibergenov, SarkytWu, Ta YeongKu-Herrera, José De JesúsWu, WangqingKujawa, JoannaWu, Wan-XiaKujawski, WojciechWu, XijiaKukla, ChristianWu, XinKukulka, David J.Wu, YiboKula, SławomirWu, YonggangKulichikhin, ValeryWu, YufeiKumar Thakur, VijayWu, YunlongKumar, AnujWu, Yu-TseKumar, AvneeshWujcik, Evan K.Kumar, BrajeshWycisk, RyszardKumar, PankajWyman, IanKumar, PawanWysokowski, MarcinKumar, PradeepXi, XuedongKumar, PrasunXia, ChangleiKumari, PunitaXia, HeshengKung, Yu-ChunXia, WenjieKunioka, MasaoXiang, ChunhuiKunitake, MasashiXiang, YuanKunnev, DimiterXiao, BoqiKunsági-Máté, SándorXiao, ChunshengKuo, ShiaoweiXiao, DunhuiKuo, Tsung-RongXiao, KaidaKurcok, PiotrXiao, KangKurek, MateuszXiao, WeiKushwah, VarunXiao, YueKusior, AnnaXiao, ZhigangKuzhir, PolinaXie, HongfengKuzhir, Polina P.Xin, HaohuiKuzmanovic, MajaXu, AirongKuznetsov, Maxim L.Xu, BaipingKwaśniewska, AnitaXu, ChungangKwiatkowska, MagdalenaXu, FengKwon, Jae SungXu, Jia-ZhuangKwon, Oh SeokXu, JinyangKwon, Seong JungXu, JunKwon, Young-WanXu, KangKyritsis, ApostolosXu, RongqingKyzas, George Z.Xu, ShengyongKyzioł, AgnieszkaXu, TaoLa Deda, MassimoXu, WentaoLa Mantia, Francesco PaoloXu, ZhiweiLa Spada, LuigiXuan, ShouhuLa, Duc DuongXue, JiajiaLa, Thanh-GiangXue, XiaoqiangLacabanne, ColetteYablonsky, Gregory S.Lach, RalfYadav, MithileshLade, Harshad S.Yadav, SushmaLadero, MiguelYadavalli, Nataraja SekharLaet, LarsYalcinkaya, FatmaLafont, UgoYamaguchi, TetsuoLagoa, RicardoYan, DadongLahem, DrissYan, DayunLai, Gwo-SungYan, Ding-XiangLai, Jui-YangYan, HongLai, Wing-FuYan, JinlongLai, YuekunYan, RuosiLaity, Peter R.Yan, ShoukeLajeunesse, Dennis RichardYang, ChengLamura, AntonioYang, Cheol-MinLan, Ting-HsunYang, HaifengLandro, L. DiYang, HsiharngLang, LihuiYang, JianxiaoLange, HeikoYang, JingLanger, BeateYang, RuihuaLanger, Jerzy J.Yang, ShengLangowski, Horst-ChristianYang, Ta-ILanzi, MassimilianoYang, Teng-ChunŁapienis, GrzegorzYang, WeiŁapiński, AndrzejYang, WeiqingŁapiński, MarcinYang, XiangliangLapkowski, MieczyslawYang, XiaodengLappan, UweYang, XuanLaranjo, MafaldaYang, YangLara-Sanchez, AgustinYang, YunzeLarrañaga, AitorYang, ZhengjinLarson, GöranYang, ZhenshengLaskowski, ŁukaszYang, ZhujinLatos-Brozio, MalgorzataYankova, RumyanaLau, Woei JyeYao, XiaohuLavorgna, MarinoYao, XuefengLázár, IstvánYao, ZhitongLazzara, GiuseppeYao, Zi-JianLe Gac, Pierre-YvesYaraki, Mohammad TavakkoliLe, Vinh TungYasin, SohailLe, XiaoxiaYazawa, KenjiroLeal, João PauloYazdani Sarvestani, HamidrezaLeclère, PhilippeYazdi, ImanLecomte, PhilippeYazdimamaghani, MostafaLedwon, PrzemyslawYe, QiangLee, Bae HoonYeager, John D.Lee, Bor-ShiunnYeh, Jui-MingLee, BunyeoulYeh, Ting-fengLee, ChangguYeom, EunseopLee, CharlesYeong, Wai YeeLee, Chia-HungYi, WeimingLee, Chih-HaoYi, YunjiLee, Ching HaoYifeng, LingLee, Dai-SooYilmaz, Catalina Natalia CheaburuLee, DonghyunYilmaz, MehmetLee, Hyang MooYin, HongyaoLee, JaehanYin, JieLee, JaehongYin, YanLee, JaewooYo, TanakaLee, Jea UkYokota, HiroshiLee, JechanYola, Prof. Mehmet L.Lee, Jin WooYong, Kar WeyLee, Jong BongYoo, Dong JinLee, JongsuYoo, Seung HwaLee, Ju-HyuckYoon, Do YeungLee, JunseongYoon, Myung-HanLee, JunseopYoshida, EriLee, Li-TingYoshida, HiroakiLee, Mei-hwaYoshida, KentaroLee, Min WookYoshihara, HiroshiLee, Rong-HoYoshihara, KumikoLee, Sang JinYou, FengLee, Seok WooYou, Jae BemLee, Sung SikYou, JungmokLee, SzetsenYou, ZhengweiLee, Wen-YaYounes, HusamLee, Xin JiatYourdkhani, MostafaLehocký, MariánYousef, SamyLei, GangYoussry, MohamedLeitão, CátiaYu, BinLeiza, JoserraYu, BingranLejcuś, KrzysztofYu, DaweiLemli, BeataYu, Deng-GuangLemos, FranciscoYu, DingshanLenzi, MarceloYu, HaifengLeón, OriettaYu, HaipengLeonardi, FrancescaYu, HuayangLeone, GemmaYu, JaecheulLepoittevin, BénédicteYu, JianLeporini, MariellaYu, JiayiLesov, IvanYu, JingangLeszczyńska, AgnieszkaYu, JunrongLettieri, MariateresaYu, KejingLettieri, StefanoYu, LeixiaoLevchik, SergejYu, LuLevin, Oleg VladislavovichYu, Ruei-SungLewandowicz, GrażynaYu, Tzyy LeonLewandowska, KatarzynaYu, WeilingLewis, RandolphYu, Weiling (Connie)Lewis, RandyYu, YuxiLewitus, DanYuan, ChaoLezov, AlexeyYuan, DianLgaz, HassaneYuan, ShushanLi, AnlongYue, ShengyingLI, BangjingYuen, Jonathan D.Li, ChunhuiYuk, HyunwooLi, DegangYun, Hyun DoLi, FenghuaYunis, RuhamahLi, HaolongYusa, Shin-ichiLi, HauyiYvergnaux, FlorentLi, HeZabierowski, PiotrLi, HongdaZabihi, OmidLi, HongzhouZacharis, Constantinos K.Li, HuZafar, FahminaLi, HuiZafar, MuhammadLi, Jiao JiaoZafar, Muhammad SohailLi, JiashenZagklis, DimitrisLi, JinZaharescu, TraianLi, JingpengZaharia, CatalinLi, JiushengZając, GrzegorzLi, JunhuiZambrano, Maria De La LuzLi, JunrongZampieri, PaoloLi, JunshengZanetti, MarcoLi, LiZanjanijam, Ali RezaLi, LiangbinZanuy, CarlosLi, LingweiZapata, PaulaLi, MeichunZappia, StefaniaLi, MengfangZarejousheghani, MashaalahLi, MingdaZarras, PeterLi, MingzhongZarrelli, MauroLi, NanZarrintaj, PayamLi, NanwenZarzar, Lauren D.Li, ShengZavyalova, Elena G.Li, ShibenZdanowski, MaciejLi, ShiqiZdyb, AgataLi, ShujunZechel, StefanLi, Ting-TingZedler, ŁukaszLi, WeiZeidi, MahdiLi, XiangŻelaziński, TomaszLi, XiangmeiZelenovskiy, Pavel S.Li, XingZelikman, EvgeniLi, XinghuaZelinka, Samuel LLi, YahongZelkó, RománaLi, YangZeng, HaoLi, YingZeng, HuangLi, YiwenZeng, Jun-JieLi, YongjinZeng, LinLi, YuanZeng, MinxiangLi, YuanzuoZeng, SongshanLi, YueZeng, XingrongLi, YuzhanZeng, YongLi, ZhaohuiZha, R. HelenLi, ZhengqiangZhai, TianruiLi, ZhiZhan, XiaohongLi, ZhonghaoZhang, ChaoLi, ZhouZhang, ChiLiaghat, GholamhosseinZhang, ChunjingLiang, JianwenZhang, ChunyongLiang, KunZhang, DouLiang, WenyanZhang, FanLiang, XingguoZhang, GuangyanLiao, ChengzhuZhang, GuoqiangLiao, Hung-JuZhang, GuoyinLiao, Shu-ChuanZhang, HailiangLiao, Ying-ChihZhang, HanLiarte, SergioZhang, HongboLignola, Gian PieroZhang, HuiliangLikodimos, VlassiosZhang, JieLim, Chung-SunZhang, JunhuaLim, Dong ChanZhang, JunzengLim, Ki-TaekZhang, KaihuanLima, Rui A.Zhang, KaiyueLima, SofiaZhang, Ke-QinLin, Cheng-TeZhang, LanyingLin, Chia-HerZhang, LiqiangLin, Chia-HuaZhang, LiqunLin, Chien-hongZhang, MengLin, Ching HsuanZhang, NanLin, Chun-PingZhang, PengfeiLin, Dan JaeZhang, QiangqiangLin, HuaijunZhang, QianqianLin, Hung-YinZhang, QichunLin, JiahongZhang, QijinLin, JianZhang, RuoyuLin, Tzu-EnZhang, Shuang-QingLin, WeifengZhang, Shui-dongLin, Yang-weiZhang, ShuyeLin, YuankunZhang, WeiLin, ZhiweiZhang, WenLin, Zuan-TaoZhang, WenjiaoLinares, JoseZhang, WenxuLing, JunZhang, WujieLins, RodrigoZhang, XiaoningLins, Vanessa De Freitas CunhaZhang, XiaoyanLinul, EmanoilZhang, XinLionetto, FrancescaZhang, XinxinLiou, Guey-ShengZhang, XuefengLiparoti, SaraZHANG, YILis Arias, Manuel J.Zhang, YileiLisiecki, AleksanderZhang, YongqinLisnevskaya, InnaZhang, YuLisuzzo, LorenzoZhang, ZhenLiszkowska, JoannaZhang, ZhienLiu, BaiquanZhang, ZhishengLiu, BaojiangZhang, ZhiyangLiu, BoZhang, ZhizhouLiu, Chao-LinZhang, ZongtaoLiu, Cheuk LunZhao, ChengjiLiu, DaojunZhao, DonglinLiu, FangZhao, FuliLiu, FeiZhao, HanyingLiu, GuangleiZhao, HongLiu, GuichengZhao, JieLiu, GuojunZhao, LianfengLiu, HangZhao, LingLiu, HongshunZhao, RongjunLiu, HongyuZhao, XinluoLiu, HongzhiZhao, YanchuanLiu, Hsia-WeiZhao, YapuLiu, Hua-MinZhao, YongLiu, JiZheng, HuandaLiu, JialeiZheng, SixunLiu, JianlinZheng, XintingLiu, JunZheng, YangongLiu, JunyiZhong, LinxinLiu, KegangZhou, AoLiu, MengmengZhou, BinbinLiu, MinghuaZhou, ChangluLiu, PengfeiZhou, DingyingLiu, PengqingZhou, DongfangLiu, RuZhou, GenghengLiu, ShumeiZhou, JiajingLiu, TianyuZhou, JianhuaLiu, WendiZhou, KunLiu, WenxiaZhou, LiLiu, Xian HuZhou, ShengtaiLiu, XianhuZhou, XinglinLiu, XiaoboZhou, XingpingLiu, XiaoqingZhou, XinxinLiu, XiuhuaZhou, XuLiu, XuqingZhou, YaoLiu, YangZhou, Ying-GuoLiu, YaodongZhou, YongchaoLiu, YijiangZhou, ZheLiu, Ying-LingZhou, ZhuxianLiu, YingtaoZhou, ZiyanLiu, YitaoZhu, BowenLiu, YizhiZhu, CaihongLiu, YongZhu, ChongyuLiu, YuxinZhu, DazhangLiu, ZhiZhu, HuieLiu, ZhitianZhu, JieLiu, ZhixiaZhu, LeiLiu, ZitongZhu, LiminLizal, FrantisekZhu, LingxiangLizardi-Mendoza, JaimeZhu, TianyuLizundia, ErlantzZhu, XiangweiLo Faro, Maria JosèZhu, YishenLobo, AndersonZhu, ZhiyuanLocatelli, MarcelloZhu, ZijieLomonaco, DiegoZhuo, YizhiLongana, Marco L.Ziąbka, MagdalenaLongo, AngelaZiats, Nicholas P.Loontjens, Ton J. A.Zic, MarkLópez De Dicastillo, AnaŽic, MarkLopez Rodilla, Jesus M.Zille, AndreaLopez, Carlos GonzalezZimmermann, FelixLopez, FrancescoZimoch-Korzycka, AnnaLópez, Kelly Johana FigueroaZinchenko, AnatolyLópez, RobertoZivaljic, NikolinaLópez-Cela, Juan JoséZmozinski, Ariane V.Lopez-Cuesta, José-MarieZodrow, Katherine R.López-López, ManuelŻołek-Tryznowska, ZuzannaLopez-Manchado, Miguel AngelZubitur, Manoli M.López-Ramírez, SimónZukowski, WitoldLoppinet, BenoitZumelzu, ErnestoLorandi, FrancescaZuo, JianLorenz, AlexanderZuorro, AntonioLoretz, BrigittaZuverza-Mena, NubiaŁoś, Szymon


